# Electrocatalytic Water Oxidation: An Overview With an Example of Translation From Lab to Market

**DOI:** 10.3389/fchem.2022.861604

**Published:** 2022-05-11

**Authors:** Rakesh Sen, Supriya Das, Aritra Nath, Priyanka Maharana, Pradipta Kar, Francis Verpoort, Pei Liang, Soumyajit Roy

**Affiliations:** ^1^ Eco-Friendly Applied Materials Laboratory (EFAML), Department of Chemical Sciences, Materials Science Centre, Indian Institute of Science Education and Research- Kolkata, Kolkata, India; ^2^ Solaire Initiative Private Limited, Bhubaneshwar and Kolkata, India; ^3^ State Key Laboratory of Advanced Technology for Materials Synthesis and Processing, Wuhan University of Technology, Wuhan, China; ^4^ Center for Environmental and Energy Research, Ghent University Global Campus, Incheon, South Korea

**Keywords:** water oxidation reaction, oxygen evolution reactions, surface modification of electrodes, oxygen generator, industrial applications of water oxidation, lab to market

## Abstract

Water oxidation has become very popular due to its prime role in water splitting and metal–air batteries. Thus, the development of efficient, abundant, and economical catalysts, as well as electrode design, is very demanding today. In this review, we have discussed the principles of electrocatalytic water oxidation reaction (WOR), the electrocatalyst and electrode design strategies for the most efficient results, and recent advancement in the oxygen evolution reaction (OER) catalyst design. Finally, we have discussed the use of OER in the Oxygen Maker (OM) design with the example of OM REDOX by Solaire Initiative Private Ltd. The review clearly summarizes the future directions and applications for sustainable energy utilization with the help of water splitting and the way forward to develop better cell designs with electrodes and catalysts for practical applications. We hope this review will offer a basic understanding of the OER process and WOR in general along with the standard parameters to evaluate the performance and encourage more WOR-based profound innovations to make their way from the lab to the market following the example of OM REDOX.

## Introduction

In 1671, scientist Robert Boyle first produced hydrogen gas by conducting the reaction between iron lings and dilute acids. Later, the research work of British Chemist Henry Cavendish and French Chemist Antoine Lavoisier led to the discovery of hydrogen gas in 1783. On the other hand, in 1771–1772, Swedish Chemist Carl Wilhelm Scheele first produced oxygen gas by heating mercuric oxide, potassium nitrate, and some other nitrates. In 1774, British chemist Joseph Priestly discovered oxygen independently, and later, in 1775–1780, Antoine Lavoisier explained the role of this gas in combustion and named it “Oxygen.” Both of these gases are very much important for our daily lives. In the last few decades, hydrogen has given importance as a potentially sustainable and renewable energy source to meet the rapidly increasing demand for energy due to the dramatic rise in world population, industrialization, and worldwide economic growth. Generally, carbon-based fossil fuels are used to meet such ever-increasing demand for energy, but these fuels are non-sustainable and have limited reserves. Massive fossil fuel combustion also causes a large amount of CO_2_ and other greenhouse gas emissions. However, the burning of Hydrogen gas with oxygen in fuel cells almost leads to zero emissions (Tang et al., 2021). That’s why, for having high mass-energy density and for being an environment-friendly clean energy carrier, hydrogen gas has been considered as an alternative to fossil fuels. Similarly, for being a strong oxidizing agent, having an important role in respiration, combustion processes, oxygen has its broad application in different industries like chemical and petrochemical industry, metal industry, oil, and gas industry, and it is also used in fish farming, glass manufacture, waste management, and oxygen therapy. Especially, during the COVID-19 pandemic, we have realized the huge demand for oxygen for medical oxygen therapy to treat the patients having low oxygen saturation. Electrochemical water splitting is one of the most promising and widespread sustainable pollution-free approaches for producing hydrogen and oxygen. In the water electrolysis process, electrical power is used to dissociate water molecules into hydrogen gas at the cathode side and oxygen gas at the anode side. To overcome different activation barriers related to these reactions, excess energy in the form of “overpotential” is required for the electrocatalysis of pure water. That is why an electrolyte (an acid or a base or a salt) is added to it, and different electrocatalysts are used to increase the efficiency of water electrolysis by lowering the “overpotential.” This electrochemical water splitting reaction has two components—Hydrogen Evolution Reaction (HER) and Oxygen Evolution Reaction (OER). HER is the reaction where hydrogen (H_2_) is generated *via* the reduction of either proton (H^+^) or H_2_O at the cathode depending on the pH of the electrolyte, and OER is the reaction where oxygen (O_2_) is generated *via* the oxidation of either the hydroxyl ion (OH^−^) or H_2_O at the anode depending on the pH of the electrolyte. Although the water splitting electrolysis is straightforward and environment-friendly, the large-scale application of this process for the production of hydrogen and oxygen is still absent today due to the low efficiency and high cost of production. Therefore, the development of efficient, low-cost, earth-abundant electrocatalysts suitable for different mediums (acidic, neutral, or alkaline) having high catalytic activity, long-term stability is highly desirable for efficient production of hydrogen and oxygen gas from water splitting to meet the commercial-scale demand of H_2_ and O_2_ since the electrocatalysts help to minimize the overpotentials for HERs and OERs. The performance of an electrocatalyst for electrochemical water oxidation depends on three key factors—1) Activity, 2) Stability, and 3) Efficiency. In this paper, the activity of electrocatalysts is explained in terms of overpotential, Tafel slope, and exchange current density. ECSA (Electrochemically Active Surface Area) is also another parameter that influences the catalytic activity of the electrocatalysts. Stability is one of the important parameters for describing the ability of an electrocatalyst to maintain its original activity over a long range of time. It can be assessed by recording the changes of the overpotential at a particular current density or by recording the variation of current density under a fixed applied overpotential, over a period of time. Another parameter that plays a significant role in evaluating the performance of an electrocatalyst is its efficiency, which is characterized by Faradaic efficiency and turn-over frequency. How these factors influence the performance of electrocatalysts are mentioned in the later section of this paper. The role of electrocatalysts in the electrochemical water-splitting reaction is another focal point of this review. In this paper, we have discussed the design strategies and the catalytic properties of recently developed OER electrocatalysts that are suitable for different mediums (acidic, neutral, or alkaline). Along with the knowledge about several efficient electrocatalysts for water-splitting reactions, it is also very much important to know their practical applications. The applications of electrocatalytic water oxidation in several fields—oxygen generator, spacecraft, Fuel Cell Electric Vehicle (FCEV) technologies, nuclear submarines, etc.—are worth mentioning. Therefore, the discussion of design strategies, electrocatalytic properties, of such electrocatalysts in this review paper is expected to lead to new advances in developing active, stable, efficient, low-cost HER, and OER electrocatalysts for the mass commercialization of water splitting-based hydrogen and oxygen production. Finally, it refers to a product developed in our start-up by us: OM REDOX.

## Principles of Electrocatalytic Water Oxidation Reaction

From the thermodynamic point of view, the water splitting reaction is an endergonic process. However, this non-spontaneous reaction can be done by providing external energy to the system, such as electricity. That’s why electrical power is needed for water electrolysis to dissociate water molecules into hydrogen gas at the cathode and oxygen gas at the anode. To overcome different activation barriers, excess energy in the form of “overpotential” is required for the electrocatalysis of pure water. The electrical conductivity of pure water is significantly less. Therefore, an electrolyte (an acid or a base or a salt) is added to it, and different electrocatalysts are used to increase the efficiency of water electrolysis by lowering the “overpotential.”

**FIGURE 1:**
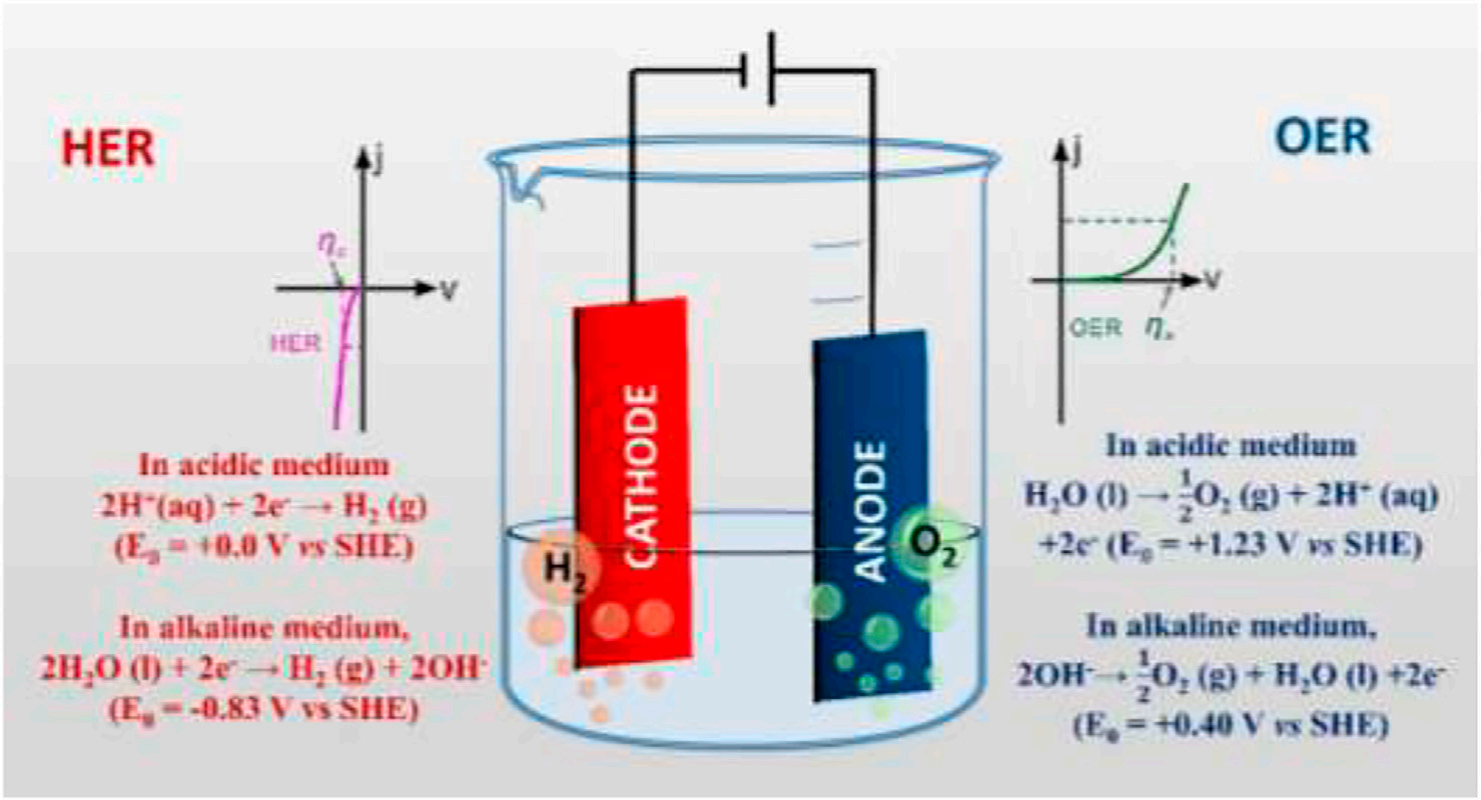
Schematic illustration of the electrochemical water-splitting reaction and related reaction kinetics.

In the acidic medium (pH < 7), excess H^+^ ions, present in the solution, move towards the cathode and are reduced to hydrogen gas, while the water molecules are oxidized at the anode to form oxygen gas. Therefore, the water electrolysis process that occurs at the surface of the electrodes in an acidic medium can be described by the following half-cell reactions (Santos et al., 2013); (Figure 1):

In acidic medium,

$$At\;Cathode:2H^{+}\left(aq\right)2e^{-}\to H_{2}\left(g\right)\left(E_{0}=+0.0VvsSHE\right)$$ (1)

$$At\;Anode:H_{2}O\left(1\right)\to \frac{1}{2}O_{2}\left(g\right)+2H^{+}\left(aq\right)+2e^{-}\left(E_{0}=+1.23VvsSHE\right)$$ (2)

$$Net\;reaction:H_{2}O\left(1\right)\to H_{2}\left(g\right)+\frac{1}{2}O_{2}\left(g\right)\left(E_{0}=-1.23VvsSHE\right)$$ (3)

However, in an alkaline medium (pH > 7), additional hydroxyl ions, present in the solution, release their electrons to anode during water electrolysis and get oxidized to form oxygen gas, while the water molecules are reduced at the cathode and generate hydrogen gas. The water electrolysis process that occurs at the surface of the electrodes in an alkaline medium can be described by the following half-cell reactions (Santos et al., 2013); (Figure 1).

In an alkaline medium,

$$At\;Cathode:2H_{2}O\left(1\right)+2e^{-}\to H_{2}\left(g\right)+2OH^{-}\left(E_{0}=-0.83VvsSHE\right)$$ (4)

$$At\;Anode:2OH^{-}\to \frac{1}{2}O_{2}\left(g\right)+2H_{2}O\left(1\right)+2e^{-}\left(E_{0}=+0.40VvsSHE\right)$$ (5)

$$Net\;reaction:H_{2}O\left(1\right)\to H_{2}\left(g\right)+\frac{1}{2}O_{2}\left(g\right)\left(E_{0}=-1.23VvsSHE\right)$$ (6)

Under the standard condition (ambient temperature and pressure), the Gibbs free energy and the enthalpy of the water-splitting reaction are ΔG^0^ = 237 kJ mol^−1^ and ΔH^0^ = 286 kJ mol^−1^, respectively. The thermodynamic standard cell potential for water-splitting reaction can be calculated by using the following equation:

$$\Delta G^{0}=nFE^{0}$$ (7)

In the above equation, ΔG^0^ is the standard Gibbs free energy, n is the number of electrons, F is Faraday’s constant, and *E* ^0^ is the standard cell potential. Using Equation 7, the theoretical reversible voltage required for dissociating a water molecule into hydrogen and oxygen gas is calculated as 1.23 V at 25°C, in both acidic and alkaline mediums (Shinagawa and Takanabe, 2017). However, in practice, to overwhelm the activation barrier of reaction, more than 1.8 V is required. This substantial overpotential for the water-splitting reactions emerges because of the sluggish transfer kinetics of the four-electron transfer in the anodic oxidation reaction and the two-electron transfer in the cathode reduction reaction (Li et al., 2020). The Nernst equation of water electrolysis can be expressed as (Shen et al., 2011)

$$E_{cell\;}^{0}=1.23-0.9\times 10^{-3}\left(T-298\right)+\frac{RT}{4F}\ln\left(\frac{p_{H_{2}^{2}}\cdot p_{O_{2}}}{p_{H_{2}O}}\right).$$ (8)

In the above equation, *p*_*H*2_, *p*_*O*2_, and *p*_*H*2*O*_ are the partial pressures of hydrogen, oxygen, and water vapor, respectively, and T refers to the temperature in K (Shen et al., 2011). The Nernst equation enables us to determine the cell potential for water splitting reaction under non-standard conditions (at reaction condition). This water dissociation potential is influenced by the catalyst activity of electrodes. Moreover, the electrochemical properties of the electrodes influence the mechanism of the water-splitting reaction. The value of water dissociation potential depends on it (Shen et al., 2011). As mentioned earlier, high overpotential is one of the main reasons for the sluggish kinetics of the water-splitting reaction. This kinetic effect of water electrolysis can be expressed with the Butler–Volmer equation *via* the activation overpotential of the cathode and anode. This equation is as follows (Shen et al., 2011):

$$\eta_{act\;,i}=\frac{RT}{F}\sinh^{-1}\left(\frac{J}{2J_{0,i}}\right)=\frac{RT}{F}\ln\left[\frac{J}{2J_{0,i}}+\sqrt{{\left(\frac{J}{2J_{0,i}}\right)}^{2}+1}\right],\;where\;,i=a,c$$ (9)

In Equation 9, $\eta_{act}$ is the activational overpotential of electrode i (i = *a*, *c*), where “*a*” and “*c*” subscripts represent the anode and cathode, respectively, *J* is the operating current density, *J*_0_ is the exchange current density, and *R* is the ideal gas constant.

The exchange current density can be expressed as the following equation (Shen et al., 2011):

$$J_{0,i}=J_{i}^{ref\;}\exp\left(\frac{E_{act,i}}{RT}\right),i=a,c,$$ (10)

where $J_{i}^{ref\;}$ is the pre-exponential factors and $E_{act,i}$ is the activation energy.

Generally, in the electrochemical water oxidation process, the activation overpotentials of the cathode and anode are different from each other, which means that they have different catalytic activities. However, the ratio of electrochemical reaction rate on the cathode to the anode or the rate of product formation at both the electrodes does not depend on their catalytic activities. Rather, it depends on the stoichiometric ratio as the current passing through the cathode and anode is always the same in the electrolysis process. Therefore, the rate of production of hydrogen and oxygen can be expressed with Equations 11, 12, respectively (Shen et al., 2011),

$$N_{H_{2}}=\frac{JA}{2F}$$ (11)

$$N_{O_{2}}=\frac{JA}{4F}$$ (12)

The rate of hydrogen and oxygen gas production is also influenced by the efficiency of water electrolysis. The efficiency of electrolysis depends on several factors. It depends on the availability of the cations and anions in the solution, their mobility rate to reach the electrode, and the required activation energy to transfer electrons from the electrode to the electrolyte ions. In addition to these, the wettability of the electrocatalyst with electrolyte and the rapid desorption of the bubbles generated on the electrodes is very important to increase the efficiency of water-dissociation reactions. Because the breaking of the generated gas bubbles from the electrode surface sometimes becomes difficult, and as a result, the electrolyte cannot easily diffuse to access the interface of the electrolyte or catalyst. Therefore, hydrophilicity and aerophobicity are considered two important facets of electrodes to perform an efficient and stable water electrolysis process. Hence, developing efficient, low-cost, stable, earth-abundant electrocatalysts for water-splitting reactions is imperative (Li et al., 2020).

## Electrode Design in Electrocatalytic Reaction Involving Water Oxidation

The simplest electrochemical cells can consist of anode and cathode immersed in an electrolyte in a glass beaker, split *via* porous separator or membrane. With the increasing need, the complexity also increases and starts including a series of complex components, including electrode plates, housing, separators, and gaskets. The modern cells now comprise new features such as well-spaced threaded fastenings for compression (seen in the electrocell, the FM01-LC electrolyzer, and the microflow cell), wrenches, thumb screws for fixing and relaxing the frame (Ralph et al., 1999), inlet and outlet flow tubes ruling out the requirement for hand tools, electrode and membrane gaskets, membrane and separator, current collector, and rear and front plate assembly.

### Fabrication of Electrode Materials

Electrochemical cell components are prime candidates for 3D printers, either from polymer materials for it to be electrically insulating or stainless steel for functioning as a current collector. Diversity of materials including conducting polymers such as polypyrroles, polythiophenes (Campbell et al., 2007; de León et al., 2008), boron-doped diamond ceramics, and electrically conductive suboxides of titanium and Ebonex (Walsh and Wills, 2010) are used for electrode materials. Mature coating techniques were developed, such as plasma and vapor deposition, anodized, and electrodeposited finishes (Walsh et al., 2015). Carbon fiber polymer and metal-ceramic/polymer are also utilized as modern ceramic composites. The most widely used is a nanostructured surface on the electrode for their high activity and area properties.

Stacked cell designs require adequate distance between the cathode and anode to prevent electrical short circuits or product crossover. In this regard, porous polymer meshes and crushed glass frits are successfully applied for electrical separation.

Here, we highlight the techniques for the design of electrodes and their surface modification, but other complementary approaches also came into notice for the realization of modern electrochemical materials, such as electrospinning and electrostatic deposition. Composite fibers of carbon nanotubes and titanium nanotubes composing biomaterials like chitosan can be produced by electrospinning. Electrostatically deposited Pd/Ir catalyzed micro-fibrous carbon mats have been utilized for peroxide reduction as a 3D cathode in a borohydride fuel cell (Dechakiatkrai et al., 2009). The continued development of classical imaging tools such as scanning electron microscopy (SEM), transmission electron microscopy (TEM), atomic force microscopy (AFM), and advanced X-ray computed tomography (Arenas et al., 2018) has helped to realize the surface morphology of structured surfaces in a great extent.

### Choice of Electrolyte

In an electrochemical reaction, one should choose solvents and supporting electrolytes considering the full electrochemical mechanism and the overpotential window for the electrolyte and the solvent. Interaction between the electrolyte ions and the catalyst surface is also very important because strongly absorbing species can reduce the reaction rate drastically by blocking the catalyst sites (Perry and Denuault, 2015). Strong pH dependence and the need for proton transfer often make aqueous solutions good options for many electrochemical systems. However, the solvent window is narrow for water compared to ionic liquids and organic media. Organic electrolytes and ionic liquids (room temperature) allow reactions through an intermediate that would otherwise be oxidized in aqueous media, thus presenting a wider window (Möhle et al., 2018). They can also stabilize high-energy radical intermediates for organic electrochemical synthesis (Wiebe et al., 2018). Ionic liquids are desirable from green and environmental perspective as these are recyclable. However, they are costly and their viscosities give low mass transport (Kathiresan and Velayutham, 2015).

### Types of Electrochemical Cells

**FIGURE 2:**
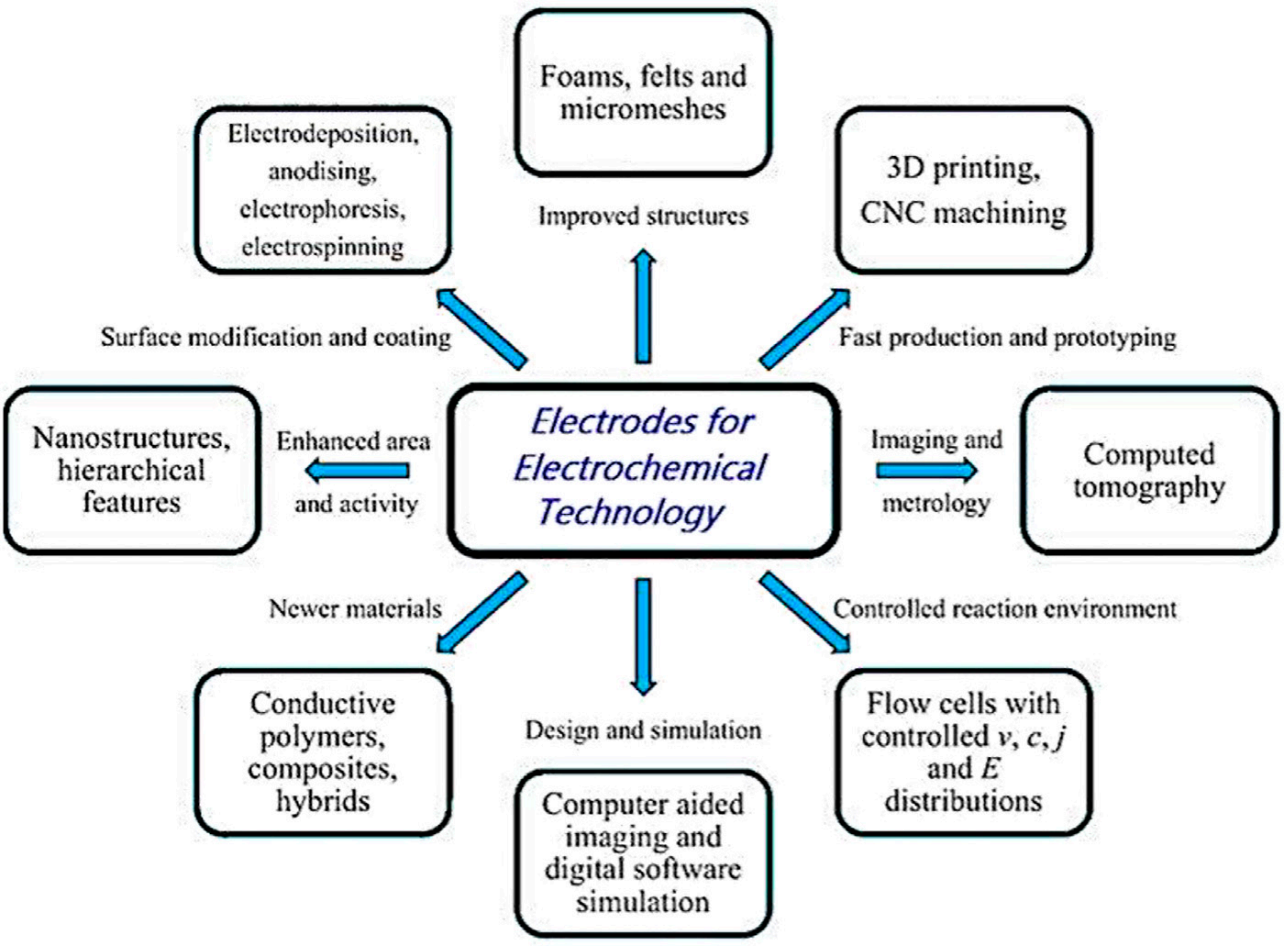
Various electrodes used in electrochemical technology, showing the diversity of materials, structures, coatings, shapes, manufacturing methods, and characterization techniques. Adopted with permission from Walsh et al. (2018).

In the 1960s, electrochemists developed engineering principles to design cells with a diverse range of electrode and cell geometries for laboratory and pilot-scale use in the 1970s (Goodridge and Scott, 2013). Such developments and principles till date are very relevant for any cell-related designing purposes. From using a diverse range of materials (e.g., metals, polymers, composites, etc.) to improving structural and surface designs (e.g., introducing nanostructures and hierarchical features or using techniques such as electrodeposition, anodizing, electrophoresis, electrospinning, etc.), several domains have been developed to improve the efficiency of electrodes for electrochemical reactions. 3D printing and lithographic techniques have been adopted for fast production and prototyping, which is aided by computer-simulated designs and a wide range of characterization techniques, from optical and SEM through to X-ray computed tomography and synchrotron radiation (catalyst) studies (Figure 2).

We have already understood that depending on the application, different types of electrodes and cell geometries are developed to optimize the conversion, current efficiency, electroactive area, mass transport, selectivity, energy transport, and efficiency. During the design, some considerations are necessary to realize their importance for the process. These are as follows: a) low-cost production by maintaining the simplicity; b) low operational cost as well as component costs such as an electrolyte and a separator; c) reliability for routine operations, cleaning and inspection; d) engineering of the reaction pathway for highest selectivity, production, mass transport, electroactive area, current and potential distribution, inter-electrode gap, and low overpotential (Fleischmann et al., 1980); e) low-pressure drop over the cell; f) ease of the product extraction; and so on.

Considering theses points, some simple cell types are already available to aid the selection of particular electrode geometry and cell design (Walsh and de Léon, 2018).When used retrospectively, this approach is very beneficial to rationalize diverse cell designs by considering their significant characteristics.

1) Filter-press cell: There are two main types: Undivided cell and Divided cell. The divided cell can be further classified into monopolar electrodes and bipolar electrodes. 2D electrodes and 3D electrodes are the two categories of bipolar electrodes. These two types of cells can be classified into commercial cells and in-house cells. Filter-press cells can also be divided into fast prototyping, such as 3D printing and conventional manufacturing by machining or molding. Both the types have applications as a commercial cell and an in-house cell.
2) More specialized cell: There are three main types: rotating electrode (e.g., rotating cylinder electrode reactor), porous electrode (e.g., packed bed or reticulated electrode reactor), and thin-film (e.g., bipolar trickle tower)

### Electrode Design: Structure and Form

**FIGURE 3:**
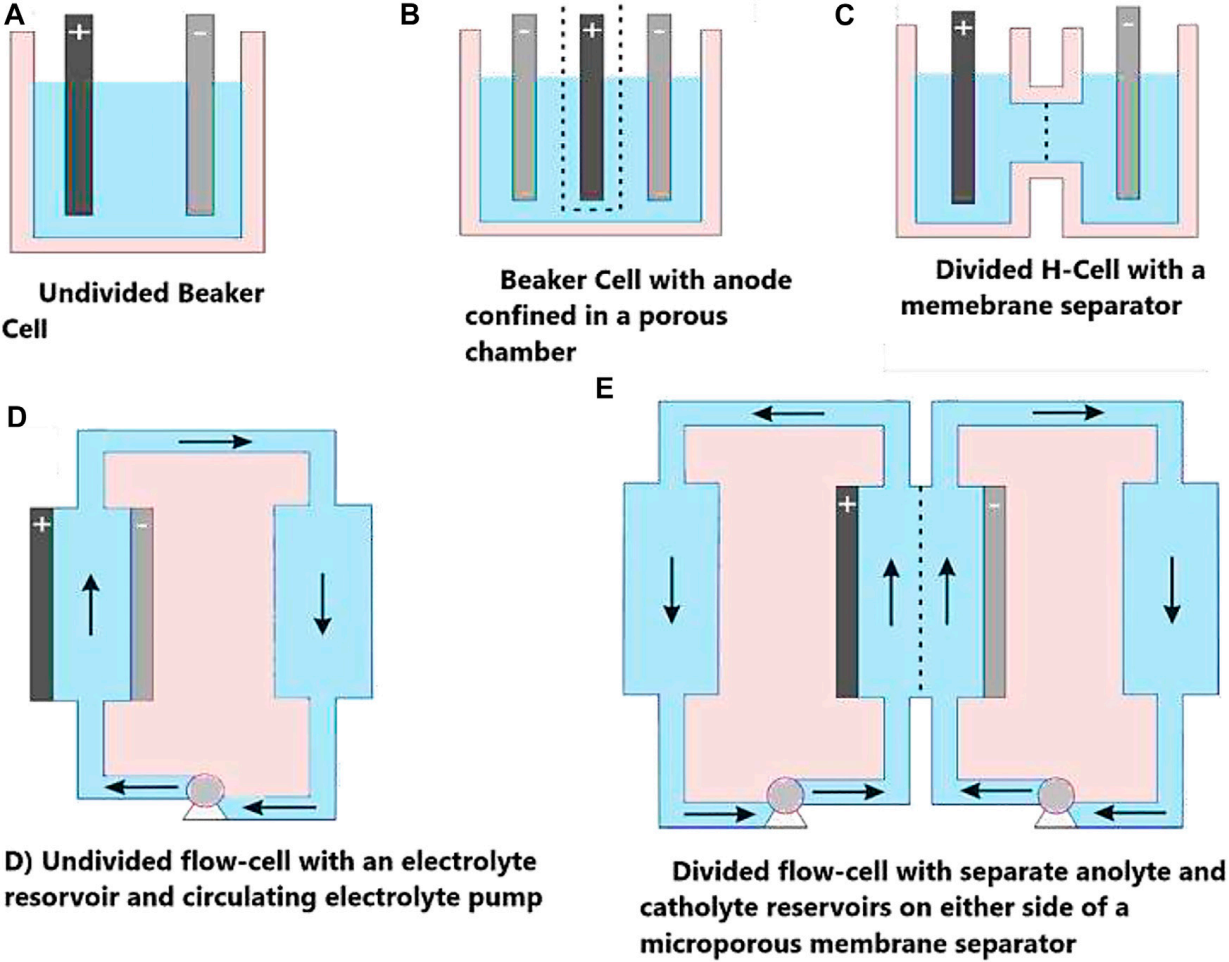
Commonly employed laboratory-scale electrochemical cells, where the solid arrows indicate the direction of electrolyte flow and dashed arrows indicate a porous separator or ion conductive membrane. Adopted with permission from Perry et al. (2020).

The scope of electrochemistry is enormous, covering anywhere from electrocatalyst studies at atomic or molecular-sized species to full-scale commercial cell rooms. The working electrodes play a critical role in determining the performance and greatly influence the reaction rate due to their different structures, conductivity, and access of the catalyst to the electrolyte. The electrode size can also be extremely diverse, laboratory electrodes ranging from 1 mm^2^ to 10 cm^2^ including microelectrodes, while pilot plant electrodes can be 100–500 cm^2^ (sometimes even 1 m^2^). Electrodes are designed for a particular use, such as electro-kinetics studies, bulk electrolysis, determination of limiting currents, or continuous reactor operation (Pletcher, 2019). The type of cell design is extensively considered elsewhere with respect to electrode shape, geometry, and electrolyte flow/electrical connections (Walsh and Reade, 1994a; b). Large scale reactions need the mass transport of reactants/products and heat transfer to/from the electrode. The design should improve sufficient mass transfer with the necessary reactants for efficient reaction progress. Thermal control is also very important as many redox reactions are exothermic in nature. Thermal control plays an important role in ensuring the stability and operational safety of the cell as active cooling may be needed in the form of heat exchange to get rid of the heat generated by the Joule effect. Figure 3 demonstrates some commonly employed laboratory scale electrochemical cells.

General considerations regarding electrodes: A) Cost is always one of the main factors, so low-cost development is always economical. B) To make the electrodes cost-efficient, an adequate lifetime of the electrodes needs good mechanical, thermal, and chemical stability along with corrosion resistance. C) A high electrical conductivity gives a uniform current distribution and low ohmic losses. Choosing materials with very low resistivity is very important since the chemical resistance of the materials increases by the use of high surface area porous materials and 3D structures. D) Electrocatalysts should be modified for higher stability and yield by providing a low overpotential for the reaction.

One of the most important points of electrode activity is its surface area. We already know the importance of achieving a high current, but increasing the surface area is necessary for efficient activity if the current density is restricted. Porous materials and 3D surfaces can offer a low-pressure drop over the flow channel and moderate density. In the case of electrodeposited solids, suitable morphology and purity should be maintained for good current efficiency (Walsh, 2001). An increase in current with the electrode area will be accompanied by ohmic and kinetic losses, leading to a more negative cell potential, and will also result in a higher capacitance,

$Cell\;potential\;difference\;U=U_{e}+\sum \left[\eta \right]+\sum j\cdot A\cdot R$ , where η = polarization, A = electrode area, and R = gas constant;

$Mass\;transport\;\lim iting\;current\;I_{L}=z\cdot F\cdot c\cdot A\cdot k_{m}=K\cdot v^{x}$ , where z = no of electron transferred, F= Faraday’s constant, c = concentration, and A = electrode area;

$Ch\arg e\;transfer\;current\;I=z\cdot F\cdot A\cdot k\cdot c\;\exp\left(\frac{\alpha \cdot z\cdot F\cdot \eta }{R\cdot T}\right)$ , where z = no of electron transferred, F= Faraday’s constant, c = concentration, A = electrode area, η = overpotential, R = gas constant, T = absolute temperature, and α= charge transfer coefficient;

Capacitance $C=\frac{q}{\Delta E}=\frac{A\cdot \int l\cdot dt}{\Delta E}$ , where q = charge, ΔE = change in potential, l = distance between the electrodes, and A = electrode area.

### Modification of Electrode Surface

The surface area of the electrodes can be increased by using porous hybrids and 3D electrodes. Porous electrodes can be arranged in two configurations: flowthrough (Alkire and Gracon, 1975) and flow-by (Alkire and Ng, 1977), where the current and electrolyte flow run parallel and perpendicular to each other, respectively. In both cases, the electrodes face each other for uniform current distribution. Reticulated vitreous carbon (RVC) (Arenas et al., 2018), foam (Mustafa et al., 2019), and carbon felt (Castañeda et al., 2017) electrodes have been extensively used for the same purpose. The strategies for modifying the electrode surface include electro-depositing high surface area, combining foam with a fibrous structure and active nano-particles on the surface. The intricate surface of the 3D electrodes enhances the mass transport of the active species to the surface and creates different resistance between the opposite points of the electrodes. This geometric influence can affect their performance by changing the potential and current distribution. The geometry of the 3D electrodes can also increase the space-time yield of the electrochemical cells compared to 2D electrodes (Arenas et al., 2016). Here, mass transport can be correlated and controlled to the pressure drop and flow dispersion to evaluate the overall cost-benefit of the electrodes. The significance of 3D and porous architecture can be realized when structures such as nanorods, nanospheres, nano-onions, networks of nanowires and nanoflowers, micro-flowers, nano walls, etc., are manufactured on flat plate electrodes or inside already three-dimensional electrodes (Egorov and O'Dwyer, 2020). The electrochemical properties can be tailored by surface treatment or deposition of electrocatalysts. One such example is the electro-deposition of Pt on the Ti plate, felt, or meshes (Arenas et al., 2019). While depositing gold nanoparticle, by sputtering, onto a nano-porous array of TiO_2_ on a Ti foil, felt or mesh surface, the hybrid surface nanostructure can be prepared by comprising “tubes within tubes” as in the incorporation of titanates via electrophoresis into an anodized microporous TiO_2_ array on a Ti foil (Low et al., 2012). The hybrid surface nanostructure can be prepared by comprising “tubes within tubes” as in the incorporation of titanates *via* electrophoresis into an anodized microporous TiO_2_ array on a Ti foil (Low et al., 2012). The surface of the electrodes can always be modified for enhanced activity to incorporate nanoparticles into it by sol-gel coating, chemical reduction of metal salts, electroless deposition, and anodic or cathodic electrodeposition. The resulting surface had an average surface roughness of ca. 100 nm. By these techniques, examples of deposited materials include (a) precious metals such as Pt, Ru, etc., by galvanic reduction of metal salts (Walsh et al., 2006); (b) Ni, Co, Pt, Sn, etc., transition metals by immersion, electroless, and cathodic deposition (Walsh and Low, 2016); (c) metal oxides such as PbO_2_ or MnO_2_ by chemical and anodic deposition (Li et al., 2011); and (d) anodic and cathodic deposition of metal–metal, metal–polymer, and metal–ceramic composites (Walsh et al., 2015).

In recent years, cell design can be assisted by computer-aided design (CAD), and computer modeling of the reaction environment such as pressure drop, electrolyte hydrodynamics, and leakage currents flowing around the electrode rather than in the ordered fashion (shunt currents) together with concentration, current, and potential distribution. Digital designing is now a breakthrough for producing multiple, improved 3D electrodes by 3D printing, which are then tested in the laboratory. The development of technology has enabled 3D printers to assess a broader range of solid materials such as auxetic polymer foams having controlled pore shape, size, and density (Critchley et al., 2013). The advancement has been seen by the production of the fast prototype laboratory Zn-Ce redox flow battery (Arenas et al., 2015). Quickly modifying and manufacturing the cell resulted in a much faster development process. It was seen that high-resolution laser curing was a more suitable technique for printing electrochemical flow cell components because melting of polymer resulted in undesired porosity and deformation due to thermal stress. Developments of 3D printing have resulted the successful manufacture of 3D porous metal electrodes. Digital imaging software allowed visualization, simulation, and modification before manufacturing.

Now coming to some disadvantages, 3D electrodes can present uneven current and potential distributions, resulting in the asymmetric electrodeposition of catalyst particles and uneven final operations. Therefore, establishing the optimal electrode thickness ensures the electrochemical activity of the covered electrode surface. Not all the surface of the thicker electrodes has the same potential, so the catalyst distribution may not be homogeneous. That is why the electrodeposition technique is the most reliably used for such proposes. There is still possibility that parts of the electrode can be inactive if the electrode is too thick or if the electrolyte species has very low concentration. However, mathematical simulation can determine the optimal thickness, such as in conductive porous electrodes. A unidirectional potential distribution under limiting current conditions can be modeled assuming plug-flow conditions and that in excess of supporting electrolyte, the conductivity changes during electrolysis are negligible (Nava et al., 2008). In practice, the potential difference between the porous electrode surface and the solutions should not be too large to ensure that hydrogen or oxygen evolution does not occur during reduction or oxidation, respectively.

**FIGURE 4:**
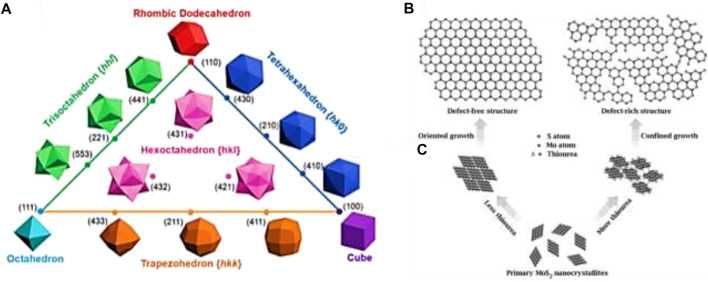
**(A)** Triangular diagram showing fcc metal polyhedrons bounded by different crystallographic facets. Reprinted (adapted) with permission from Yu et al. (2010). Copyright (2010) American Chemical Society. **(B)** Structural models of defect-free and defect-rich structures. **(C)** Synthetic pathways to obtain the above two structures. Adapted with permission from Xie et al. (2013).

Catalytic properties of nanostructure not only depend on the surface area but also on the surface facets. For a face-centered cubic (fcc) lattice structure, surface facets such as {111}, {100}, and {110} can be classified as low-index facets (Figure 4A). Nanocrystals usually form low index facets to minimize the surface energy. On the other hand, nanocrystals with high index planes show enhanced activities due to their higher surface energy and lower coordinated atoms. Thus, controlling the nucleation and growth process through synthesis can develop high index surfaces for valuable applications. As an example, the activities of high index facets of Pt, such as Pt (520), Pt (710), Pt (310), etc., with large density of atomic steps and dangling bonds, show a significantly higher formic acid oxidation rate than those of low index facets (Tian et al., 2007). Thus, the need formation of surface facets of a particular nanocrystal can be altered by introducing foreign species into the system and the absorption ability of the species on the metal surface can change the surface energy. As the particle size shows a strong correlation with the nanocrystal formation, the growth rates of various facets can be controlled by selecting appropriate surface capping reagents. Therefore, through capping agents, one can regulate the two rates (nucleation and growth rates) as well as two mechanisms (thermodynamic control and kinetic control) to obtain 1D or 2D nanostructures by inducing either anisotropic growth or epitaxial growth.

On a catalyst surface, not all sites are equally active. The active catalyst sites are regarded as low-coordinated surface atomic sites, including the low-coordinated atoms at terrace, steps, kinks, edges, corners, vertexes, vacancies, defects, holes, and pits that often favor catalytic reactions. Active sites can be strongly correlated with the surface roughness of the electrode and the number depends on the features of the surface and interface. A metal surface can generally be of two types: 1) equilibrated metal surface (EMS), which is well embedded, predominant, and stable surface; and 2) metastable metal surface (MMS), which is protruding, low-population, and high-energy surface. The MMS atoms act as the surface-active sites in general because these are prone to undergo reactions with the involved species at low overpotentials (Lertanantawong et al., 2008). Active sites are intuitively associated with the surface facets of the nanocrystal. The metal surface is enclosed by different types of atoms whose coordination numbers follow the order of face > edge > vertex with the reverse order in terms of catalytic activity. For different well-defined shapes, the variation of the surface facets can alter the proportions of various surface atoms with various coordinated states, affecting the catalytic properties thereafter. Active sites are also related to surface defects and vacancies as well as on the surface area and the particle size. Breaking chemical bonds to endow atoms with low coordinated states is beneficial to create active sites. Defect or vacancy on the basal planes of the catalyst can increase the exposure of active sites by forming cracks on the surfaces, and as a result, the electrocatalytic activity may dramatically increase (Xie et al., 2013) (Figures 4B,C).

Interfaces are formed when different phases or objects contact each other. Thus, heterogeneous catalytic reactions can be regarded as various interfacial effects. Engineering the interface between the catalyst and electrode can potentially alter the geometrical structures, and interfacial compositions, electronic structure and electron transport, Fermi levels and band alignments, proton transfer and species exchange, coordination numbers and atomic arrangements, crystal structures and dangling bonds, and selective adsorption and desorption, which all have tunable and synergistic effects during the heterogeneous electrocatalysis at the active sites of the electrode catalysts (Xia et al., 2015). For instance, the Pt_3_Y nanostructure can modify the d-band depth, surface adsorption ability, and electronic structure to improve the oxygen reduction reaction (ORR) performance (Hwang et al., 2012).

### Electrocatalyst Modified Electrodes

When an electrode such as a piece of Pt or carbon is dipped into a solution, its surface is then covered by a layer of water molecules or species present in the electrolyte solution. Such adsorbed species can often modify the electrochemical activity of the electrode. Now purposely modified electrode surfaces can produce electrodes with new and exciting properties that may form the basis of new applications of electrochemistry (Murray, 1980) as we have already discussed a bit in the case of electrolysis of water. In addition, many reactions that one would like to carry out in electrochemical cells do not occur readily at inexpensive electrode materials (e.g., carbon). Before the mid-1970s, electrochemistry was confined only with electrode materials such as C, Au, Pt, Hg, etc. Murray and co-workers initiated the field of chemically modified electrodes (CME) by taking the fundamental group transformation to SnO_2_ and Pt-OH (Murray et al., 1987). That is why catalysis is important to enhance the reaction rate by introducing suitable, stable layers to the electrode surface. By applying known chemical principles about the structure and reactivity, useful surface catalysts can be developed for individual reactions considering the cost-effectiveness and commercialization. One of such first examples is the modification of a graphite surface by irreversible adsorption of a co-facial dicobalt porphyrin dimer which allowed the reduction of oxygen under conditions where reduction was not possible on the substrate itself. The path was sensitive to the detailed structure of the attached molecule. There are four main possible routes for preparing CMEs: sorption, covalently modified carbon, redox polymer coatings (uniform), and heterogeneous multilayers (non-uniform). Physical and chemical interaction properties are utilized for sorption-based modified electrodes. Easy surface modification and functional group attachment are the chief advantages of this approach. Thiols’ self-assembled monolayer (SAM) of Au-chemisorbed is a well-known example of this aspect (Chaki and Vijayamohanan, 2002). Using a specific functional group for covalent modification of the electrode surface is also very important. For example, the glassy carbon electrode (GCE) can form attachment with the >C=O and >C−*O*− functional groups and–OH groups from oxide electrodes (Downard, 2000). Polymer-based multilayer CMEs can resolve the lack of active components on the monolayer electrode surface. There are two ways to prepare these: homogeneous (uniform) and heterogeneous (non-uniform). Non-uniform systems are created on heterogeneous supports such as zeolite, SiO_2_ (sol-gel), clay, carbon paste, epoxy resin, phosphomolybdic acid (PMo_12_), and other polymeric systems (Sadakane and Steckhan, 1998; Walcarius, 2001a; Walcarius, 2001b; Kong et al., 2001).The uniform multilayer preparation includes ionomers, inorganic polymers, redox polymers, electrochemically deposition of mediators (metals or simple metal complexes), and mediator bearing monomers (pyrrole and amine containing complexes) (Murray, 1992).

Pure organic or organometallic complexes can be physisorbed on porous carbon bases such as vitreous carbon (VCE), graphite (GE), basal plane pyrolytic graphite (BPG), and ordinary pyrolytic graphite (OPG) for the preparation of sorption-based CMEs, by simply coating the electrodes with non-aqueous solution followed by droplet evaporation. For example, the pyridine solvent was used to modify cobalt phthalocyanine on VCE with a surface coverage of ca. 3.1 × 10^–11^ mol cm^−2^ for nitrite oxidative detection (Caro et al., 2002). Carbon nanotubes (CNTs), having a honeycomb-like nano-lattice structure with cylindrical closed topology, has unique absorption abilities for special applications as catalysts, sensors, etc. (Baughman et al., 2002; Haddon, 2002). Clay modified electrodes are other CMEs that are prepared by dip-coating of the aqueous colloidal solution (Baughman et al., 2002). GCE is quite unstable for such coating due to its polished surface nature, and additional polymeric systems are required for stability improvement (Zen et al., 2002b). The stability problems of physisorbed systems can be overcome by the chemisorbed route. Due to the easy preparation procedure of the self-assembly monolayer (SAM)/Au-oriented system by simple soaking up the Au electrode in thiol (−SH) and ethanol solutions, this kind of system carries great importance in the chemisorbed studies. Some recent progress in analytical chemistry using SAM/Au is cysteine for CO oxidation (Shi et al., 2001), disulfide derivatives of vitamin B_12_ for ORR (Viana et al., 2002), etc.

The surface functional groups of the base electrode can be made to form covalent linkage either by controlling the oxidation/reduction potentials or by the synthetic route in a suitable medium. First examples of such functional group transformation on the electrode surface are ≡Sn−OH and ≡Pt−OH. The carbon surface has many alterable functionalities and is hence very suitable for covalent modification (Downard, 2000). Aryl diazonium (Allongue et al., 1997), aryl acetate, and amino (Deinhammer et al., 1994) are most viable for covalent modification than other organic entities. For example, the anthraquinone (AC)-grafted GCE surface (GC-AQ) in coupling with the Au ring disk electrode was used for the ORR (Tammeveski et al., 2001). The GC-AQ has produced a defined potential at −0.9 V (vs. SCE) in 0.1 (M) KOH with $\Gamma_{AQ}$ = 2.5 × 10^–10^ mol.*cm*−^2^. 4,5-dihydroxyAQ-2-carboxylic acid modified GCE was prepared by the simple potential electrochemical cycling method in pH = 3 solution for its use in the reductive detection of hemoglobin at −0.2 V (vs. Ag/AgCl) (Zhang et al., 2002). These were further modified by the solution phase synthetic method with exfoliating graphite (EC) powder to form covalently modified AQ-EC (Ramesh and Sampath, 2001).

Oyama and Anson first utilized the plan to modify poly (4-vinyl pyridine) (PVP) on the carbon surface for the ion exchange of ferrocyanide ions in acidic solutions (Oyama and Anson, 1980). Thus, the modification of specific ion-exchange membranes or polymers on the electrode surface is a very interesting branch in electrochemical analysis, where the system allows the ionic species to incorporate as counterions inside the galleries. The cationic exchange membrane Tosflex was applied in analytical applications that have analog backbone like Nafion (Dunsch et al., 1990), as PVP and Nafion were extensively utilized for various applications. Selectivity is an essential criterion for sensors, and ionomeric systems can also be used for sensing charged species other than catalytic activity. Electrochemical depositions of monomeric units under potential cyclic conditions or by galvanostatic/potentiostatic methods opened another elegant way of preparing electrodes with homogeneous multilayer CMEs. A bare polymeric system or pure organic polymer without any metal and metal complex can successfully participate in the electron transfer reactions such as the formation of nitronium or oxonium radicals as a typical example. In this category, Nile blue was used for the ORR and oxidation of NADH (Ju and Shen, 2001). Electrochemical co-deposition is another popular area to utilize to form uniform multicomponent films. Besides organic sources as modifiers, simple metal plate electrodes can also be applicable for these applications. A disposable copper-plated screen-printed electrode (SPE) (i.e., CuSPE) was invented by Zen et al. for the detection of H_2_O_2_ (Zen et al., 2000a), which was modified further with glucose oxidase for glucose sensing (Zen et al., 2002a). Inorganic polymers are also looked at with equal interest towards potentiometric and amperometric sensing. Prussian Blue (PB), known for more than 250 years, was first utilized in electrochemistry in 1978 for various applications (Karyakin, 2001).

In heterogeneous multilayer CMEs, the solid supports are deliberately combined with the mediator system in a non-uniform way. Carbon paste electrode (CPE) is prepared simply by mixing binder paste/graphite and redox mediator and it is the most convenient to make among others. This procedure can be utilized to make enzymatic clay and zeolite-modified electrodes. One such example of this utilization is the MB, methyl viologen (MV), and benzyl viologen (BV)-modified zirconium phosphate electrode for NADH oxidation (Munteanu et al., 2001). This approach is also suitable for preparing multiple integrated systems. Lev’s group introduced carbon-ceramic composite electrodes (CCEs) as a modern technique to prepare stable multilayers (Tsionsky et al., 1994). Later, the CCE system was extremely introduced for analytical purposes (Wang et al., 1997). In the CCE preparation route, mediators were first mixed with SiO_2_ first in an appropriate ratio with alcohol and dilute HCl, sonicated for a couple of minutes after mixing it with carbon powder. Then it was converted into a suitable electrode by filling in a glass tube or by coating on conducting support. Other applications include making of an ethanol sensor out of alcohol oxidase enzyme (AOx) (Boujtita et al., 2000), a phenol sensor by the mixture of graphite, acetyl cellulose acetate (ACA) with ferrocene and *pseudomonas* bacteria (Skládal et al., 2002), an H_2_O_2_ censor by PVA, etc. (Wang and Dong, 2000). Industrially iron-enriched waste cinder (CFe*) was also reported as a useful host to form the stable hybrid {CFe*- Fe(CN)} derivatives directly inside the matrix by potential cycling with cyanometallate in pH = 2 KC/HCl solutions (Zen et al., 2000b). Hence, from these chemical modification types of the electrodes, we get to know the many potential applications already discovered for various analytical applications for multiple purposes and realized that this can play a key role for new inventions in the near future.

## Surface Engineering for Electrocatalytic Water Oxidation

Hence, after all the discussions related to electrode materials, electrode designs and geometry, and the electrocatalysts, we can describe what the ideal electrode design should be for successful water oxidation and OER. As we already know, the working electrodes play a critical role in determining the performance and greatly influence the reaction rate due to their unique structure, degree of wettability, and access of catalyst to an electrolyte. For example, flat surface electrodes like glassy carbon allow the single-way penetration of the electrolyte that limits the catalysis only on the surface of the catalyst, whereas 3D electrodes like carbon cloth allow multiple pathways for electrolyte penetration from all sides of the catalyst and involve all the materials in the catalytic reaction (Dou et al., 2016; Tahir et al., 2017).They all have advantages and disadvantages like glassy carbon electrodes are easy to handle and widely used in the literature but offer only limited access to the catalyst. Thus, in this section, the discussion will be regarding the type of electrode surface morphology and catalyst property that will make water oxidation most feasible.

### Surface Area

**FIGURE 5:**
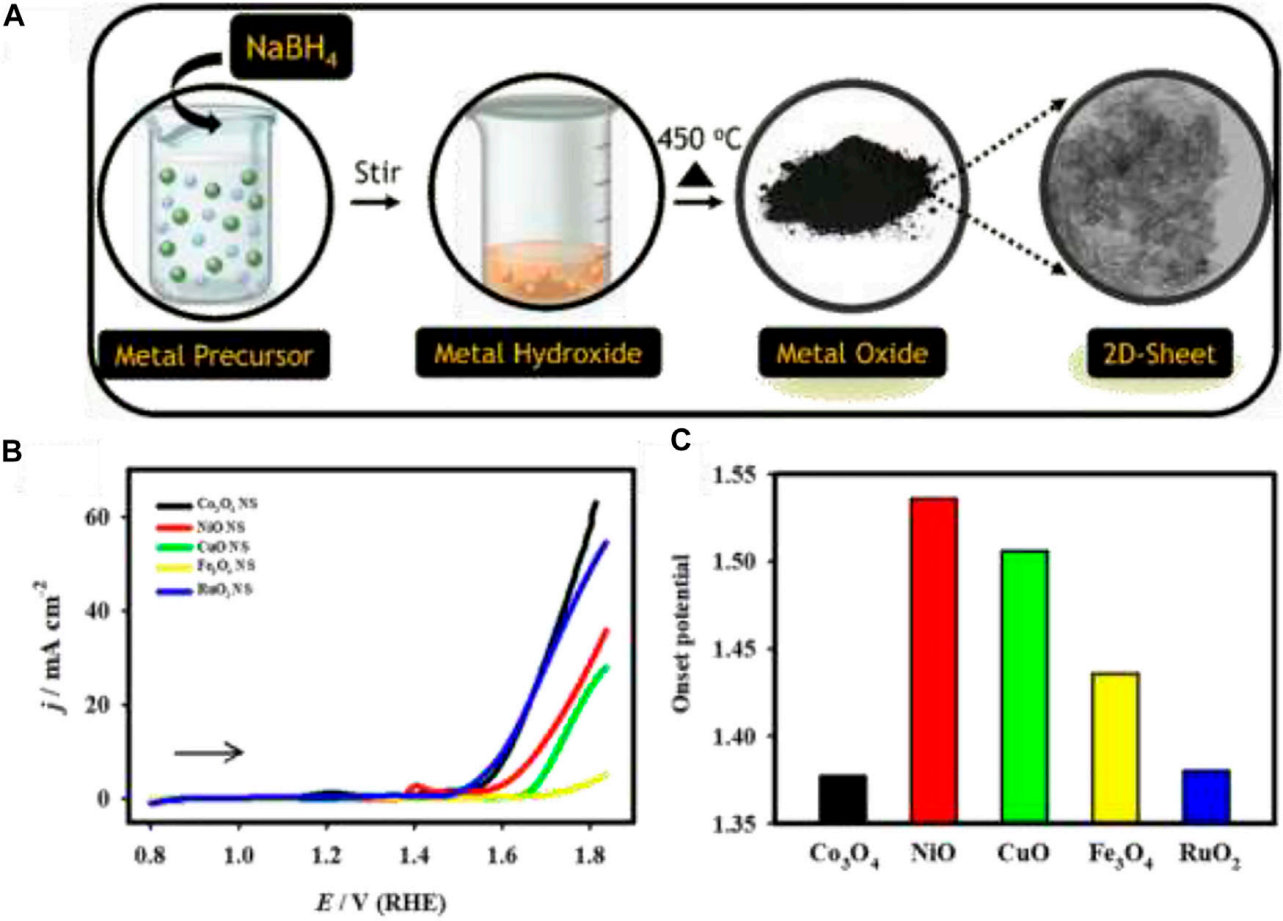
**(A)** Schematic illustration of the synthesis of 2d-metal oxide nanosheets, **(B)** LSV curves of the Co_3_O_4_ (black), NiO (red), CuO (green), Fe_3_O_4_ (yellow), and RuO_2_ (blue) electrodes recorded in 1.0 M KOH at a scan rate of 20 mV s^−1^. **(C)** Plot of the OER onset potential: Reprinted (adapted) with permission from Elakkiya and Maduraiveeran (2020). Copyright 2020 American Chemical Society.

Heterogeneous catalytic reactions, in general, occur on the surface of the electrode. Therefore, we already realized that a large surface area would be advantageous for catalytic reactions. We can apply four different methods to increase the surface area of a catalyst. Decreasing the size of the particle can dramatically boost the surface-to-volume ratio, thus increasing the effective surface area without altering the total electrode area. This is one of the most important reasons to widely use the size-control strategy in heterogeneous catalysis. If we can decrease the surface nanostructure size by 2 nm, their surface-to-volume ratio can be increased to 80%. The other approach is to synthesize catalysts into thin-layer 2D nanostructures (such as 2D nanosheets and 2D nanoplates). This also has the same effect to increase the surface-to-volume ratio of catalysts. Metal oxides and metal chalcogenides can be used for this specific purpose of 2D nanostructures for the optimized synthesis and design of the electrode, and these materials are proven to be useful to improve the surface area, active reaction sites, flexibility, and dispersion (Du et al., 2019; Elakkiya and Maduraiveeran, 2020); (Figure 5). Another approach is to increase the roughness of the catalyst surface nanostructures to form convex, concave, pilcated, or cracking rough nanostructures. These types of unique structures with morphologies analogs to flowers, dendrites, tapers, multi-pods, stars, starfishes, islands, and cactuses can naturally promote the effective surface area for catalytic performance enhancement (McCrory et al., 2013). The last way is to manufacture hollow, porous, or mesoporous nanostructures (nanotubes, nano-frameworks, nanocages, nano-boxes, and metalorganic framework) with porosities that not only can increase the number of active reaction sites and the specific surface areas but also efficiently accelerates the transportation and exchange of species of the catalyst surface (Lv et al., 2015; Wu et al., 2018). Besides all these means, 3D nano-porous structures of noble metals and their alloys can be prepared through the methods of electrodeposition, templating, annealing, de-alloying, *in-situ* synthesis, and galvanic replacement and modification for various electrocatalytic applications (Lu, 2019).

### Low-Index Facets

Surface facets influence the surface morphology and activity to a great extent due to different atomic arrangements of various facets. For example, in the case of an fcc noble-metal crystal, the low index facets generally are {111}, {100}, and {110} with their surface energies already stated as γ{111} < γ{100} < γ{110} (Fang et al., 2015). The activities of different surfaces depend on the reaction type and reaction media. For this purpose, to utilize here, we can use the acquisition of metal nanocrystals bound by different low-index facets with comparable sizes. This will allow us to investigate the facet-dependent effects free from size-effect interference. To give an example to illustrate, we can take Pd nano-crystals, which can be Pd nano-cubes enclosed by {100} facets and Pd octahedrons enclosed by {111} facets. The sizes of the nanostructures here can be controlled by applying etching techniques or reaction times. The Pd {100} surface can better activate oxygen species during reaction (Toyoshima et al., 2012); thus, this facet is significantly more active in electrode catalysis than the Pd {111} facet. Low-index facets can also be made catalytically active by tuning the particle sizes of nanocrystals on the electrode surface to alter the surface area. The size effect can influence the reaction rate, like with the shrinkage of the particle size, reaction rate and efficiency increase due to the boosted surface to volume ratio. The size control can provide an excellent platform for the investigation of size-dependent catalytic activities in various reactions for catalytic efficiency improvement, most of the times by reducing the particle size for a more exposed surface area.

Another evaluation of low-index facets includes assessing the effect of electrical contact between low-index facets and electrodes on electrocatalytic performance. Low contact resistance supports the electrode’s high efficiency despite being consist of low-index facets. Sometimes this can even outperform high-index facets despite having higher surface energy. As an example, Pt multi-cubes with {100} facets (high coverage) achieve high catalytic activity (ORR), which can be comparable to Pt multi-pods with high-index {311} facets (Ma et al., 2015).

### High-Index Facets

We have already examined that high-index facets have much higher surface energy and catalytic activity than low-index facets, which usually act as distinct catalytic active sites for breaking chemical bonds. Electrocatalysts for metal electrodes can be synthesized with well-defined morphologies and high-index facets to exhibit significantly enhanced catalytic performance. These types of nano-crystals can be classified into monometallic and multi-metallic nano-crystals. For instance, unique Pt nanocrystals with high-index facets (tetrahexahedrons/concave polyhedrons/concave nano cubes) show improved catalytic activity in many aspects (like ORR) (Luo et al., 2018). High-index facets possess a large number of steps, kinks, edges, corners, terraces, and vertexes, so they have more low-coordinated active sites on the surface to enable high electrocatalytic activities. Thus, the preparation of high-index facets can act as the solution to many electrocatalytic problems of metal nanocrystals. Oxidation etching can be used to introduce high-index facets with branched nanocrystals. The modification of etching strength can tailor several important parameters in the synthesis including, structure of seeds, the growth sites on the seeds, and the rate of atomic addition on the seeds (Xia et al., 2017). Therefore, tunable branched structures with tripods, tetrapods, hexapods, and octopods can be obtained. In the case of Pt, hexapods and octopods have higher active surface areas and roughness than those of Pt/C catalysts. Generally, these multi-pods have activity orders: octopods > hexapods > tetrapods > tripods (Ma et al., 2012). The superior electrocatalytic performance of octopods is ascribed to their high densities of stepped surfaces. Uneven anisotropic growth can also introduce high-index facets. The electrocatalytic performance of nanocrystals is usually increased in the order: octahedrons < nanowires < nanocubes < nanotapers. Nanotapers have the most densities of high-index facets, thus most active in electrochemical reactions (WANG et al., 2013a).

### Fabrication of Multi-Metallic Nanostructures

Electrocatalytic reactions can also occur at active sites on the interface besides the surface of the electrode. The interfaces are the sites relating to the species exchange, electronic structure, and charge transport. Thus, the interface design is crucial for good electrocatalytic performance. For exposed interfaces, these can also act as the active sites for the reactions. Now hybrid and alloys are two different types of multi-metallic nanostructures, having different electronic structures, atomic arrangements, functional sites, d-band depths, and compositions. By using this method, we can reduce the usage of costly metals like Pt by preparing multi-metallic nanostructures with similar or better activity (Zhang and Wöllner, 2018; Li et al., 2021). Now, we already know that hybrid materials possess high-index surfaces with high surface energy for effective catalytic activity. Thus, we can fabricate high-performance and low-cost catalytic electrodes through controllable growth of noble metals on the surface of the base electrode by simply modifying the surface and interface conditions. Alloying represents another approach to control the performance through tuning of atomic interfacing. Alloying can be used to prevent the oxidation of any metal nanostructure by mixing with it any passive metal. For example, Cu nanocrystals cannot survive from oxidation but alloying it with platinum to give Cu_x_Pt_100−x_ improve the chemical stability, catalytic activity, and positive shifted the overpotential (Zhao et al., 2015).

### 2D Materials

2D materials may have prominent geometrical, thermal, mechanical, and electrical characteristics along with excellent solubility, adhesion, and dispersion altogether. Thus, graphene-like 2D materials can be used as support to fabricate unique hybrid structures to achieve optimized electrocatalytic performance (Zhao et al., 2019). These materials can also exhibit synergistic effects. Modified 2D hybrid materials can enhance electron conductivity, promote catalyst electrode adhesion, and suppress the agglomeration and oxidation erosion of catalysts (Fang et al., 2020). Through the fast development of 2D materials, the main challenge is to synthesize them properly and later stabilize them for the application, as metal atoms have strong preference to form-closed packed crystal structures in three dimensions. Recently, development has been made to synthesize nanosheets and nanoplates of Pd, Rh, Ru, Ag, Au, Pt-Cu, etc., with catalytic and optical applications, which has increased the possibility of fabricating 2D metal structures (Saleem et al., 2013; Fan et al., 2015).

### Interfacial Polarization

Charge on the surface can affect molecular adsorption on the surface and promotes species-catalyst charge transfer, and thus is a very important parameter in this regard. Polarization can tune the surface charge state of catalysts among the other approaches adopted. An example of this kind is graphene doped with nitrogen due to the electronegativity difference of N and C. polarization can be induced by external electrical field or ferroelectric substrates and charges accumulated at the interface can be extended to the surface, modifying the surface charge state of catalysts as a surface polarization (Shao et al., 2010; Wang et al., 2010). In a hybrid structure, the contact of two different metals has different work functions, which can cause a flow of electrons from one metal to another to equilibrate the electron Fermi distribution and induce polarization at the interface. Thereafter, reducing the distance between the polarized interface and reactive surface can cause accumulation of a particular charge on the surface, which may help in water oxidation. Thus, tuning the thickness to the effective level is very important to achieve effective surface polarization (Lin et al., 2018).

### Surface-State Passivation

The high density of surface states mainly due to crystalline defects is found in many semiconductor electrodes used for water oxidation. Although these may possess good charge transfer kinetics, the surface states are generally considered detrimental for OER electrodes because these may attract the movement of electrons toward the surface and act as traps for charge recombination at the surface. This can cause a shift in the overpotential value. Therefore, these are highly needed to be passivated in order to improve the OER performance. For example, CoPi, FeNiOx have been reported to effectively passivate the surface states of hematite for better water oxidation (Du et al., 2013; Thorne et al., 2016).

### Catalytic Efficiency Preservation

The conversion or deactivation of oxygen evolution catalysts can be the major concern for long-term operation. Moreover, depositing a thick layer of catalysts may not solve this problem but can give rise to the issue of catalyst overloading and slow down the reaction due to undesired charge recombination on the surface. This problem can be solved by *in-situ* catalyst regeneration to selectively deposit new catalyst where the catalytic activity decreases due to the loss of catalyst to preserve the high catalytic efficiency of the electrode. In combination with this corrosion inhibition property, more stability can be given by high-temperature annealing enabled by doping another metal into the base electrode metal. Life span and solar to hydrogen efficiency (STH) efficiency are the most important parameters affecting the economic feasibility and energy metrics (Sathre et al., 2014; Kuang et al., 2017). Many materials used for water oxidation are unstable for water oxidation due to their surface corrosion/dissolution. One such solution to this problem is to apply water oxidation catalysts to efficiently extract charges to reduce the probability of surface oxidation by the accumulated charges (Abe et al., 2010). We can also design the reaction in such a way as to change the catalytic pathways to suppress the generation of harmful products. Still, the working electrode is partially exposed to the solution because these electrodes are either made of scattered nanoparticles or porous films permeable to the electrolyte solution. A more effective solution is to protect the electrode surface by completely separating it from solutions using dense solid films. These protective layers should ideally not allow the permeation of electrolytes, conduct holes to facilitate charge transfer, and form good quality junctions with a large barrier height. These protective layers can be prepared by atomic layer deposition (ALD) and sputtering techniques (Hu et al., 2014; Mei et al., 2014; Hu et al., 2015; Kuang et al., 2017).

## Recent Advances in Improving OER Catalysts

Electrocatalysts are catalysts used in electrochemical reactions (reactions that include at least one outer-sphere electron transfer event for the occurrence of overall chemical transformation) to facilitate the reaction process. An electrocatalyst can either perform its functions on the electrode surface or act as the electrode itself (Suen et al., 2017). Without being consumed, electrocatalysts can increase the speed of the reactions by lowering the energy barrier for the electrochemical reactions. Generally, the main function of the electrocatalyst is to adsorb reactants on the surface to generate adsorbed intermediate. This process facilitates the charge transfer between the reactant and electrode (Suen et al., 2017).Thus, electrocatalysts reduce the potential required for electrochemical transformation.

The performance of an electrocatalyst for electrochemical water oxidation can be evaluated based on three key factors—1) activity, 2) stability, and 3) efficiency. The activity of electrocatalysts can be explained in terms of overpotential, Tafel slope, and exchange current density. Polarization curves help to know about these parameters. ECSA (Electrochemically Active Surface Area) is also another parameter that influences the catalytic activity of the electrocatalysts. Stability is one of the important parameters for describing the ability of an electrocatalyst to maintain its original activity over a long range of time. It can be assessed by recording the changes of the overpotential at a particular current density or by recording the variation of current density under a fixed applied overpotential, over a period of time. Another parameter that plays a significant role in evaluating the performance of an electrocatalyst is its efficiency, which is characterized by Faradaic efficiency and turn-over frequency.

Electrochemical water splitting is a clean and sustainable way to produce both hydrogen and oxygen through the use of renewable such as solar or wind (Debe, 2012), during which the OER takes place on anode through oxidation and HER takes place on the cathode through reduction. (Wang et al., 2019). In OER, molecular oxygen is produced *via* several proton/electron coupled procedures (Busch et al., 2016).The reaction can be very sensitive to pH. Two water molecules are oxidized into an oxygen molecule (O_2_) and four protons (H^+^) basically in acidic and neutral conditions, while H_2_O and O_2_ are generated in the basic condition by the oxidation of hydroxyl groups (*OH* ^−^). As we know, a potential difference of 1.23 V is required to drive the OER reaction, according to the Nernst equation; a unit shift in pH can shift the reaction potential approximately 59 mV. OER requires the transfer of four electrons, and a kinetically favorable OER process occurs through multi-step reactions with single electron transfer in each step. Therefore, the accumulation of energy of each step makes OER kinetics very sluggish that results in large overpotential for the process. This is where the need for electrocatalyst emerges again to overcome the energy barrier by increasing the electrode activity (Gorlin and Jaramillo, 2010; McCrory et al., 2013).

### Noble Metal-Based Materials

**FIGURE 6:**
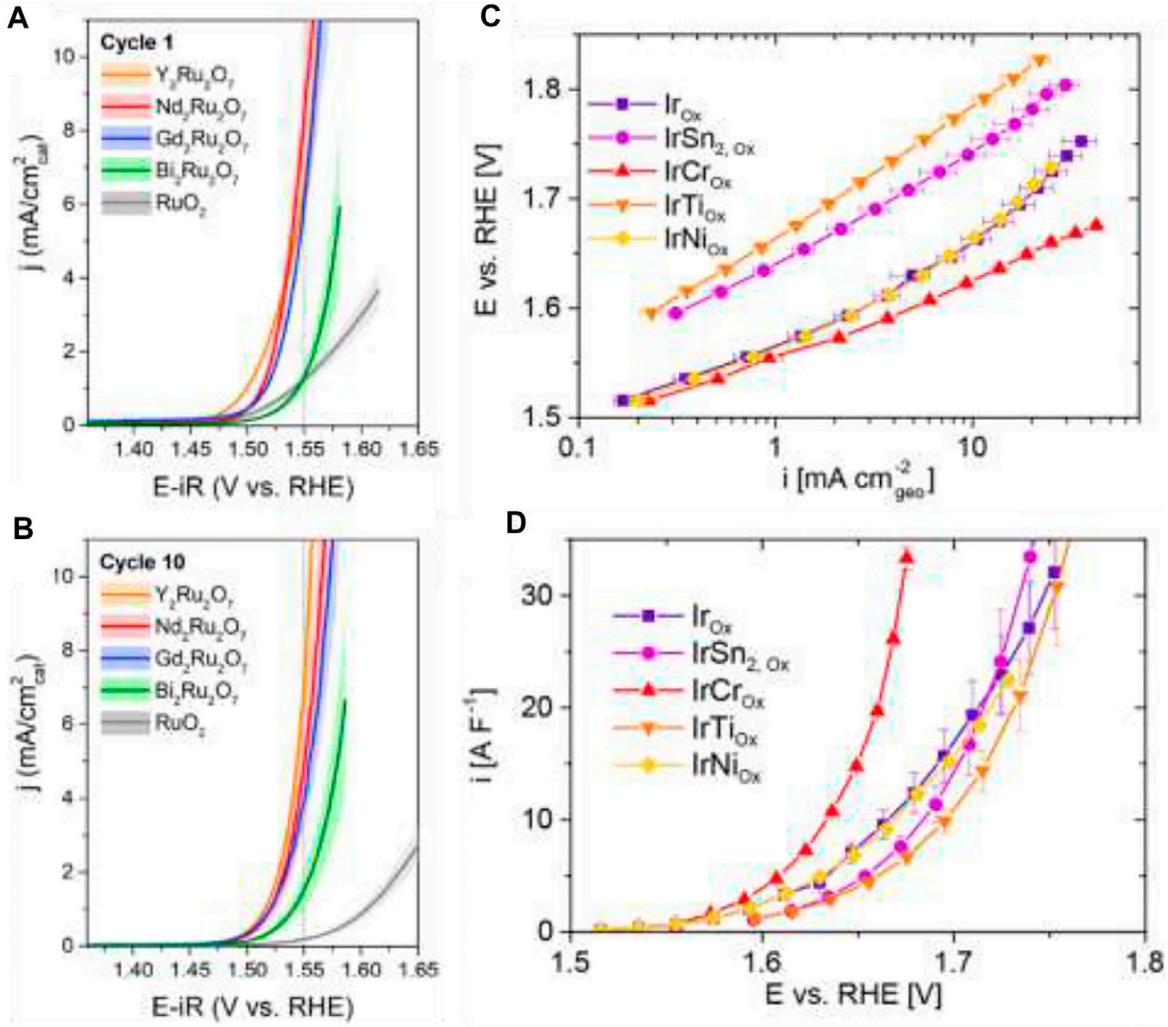
Forward sweep of first **(A)** and 10th **(B)** CV of ruthenium-based catalysts. Reprinted (adapted) with permission from Hubert et al. (2020). Copyright 2020 American Chemical Society. Electrochemical OER activity of electrochemically oxidized Ir, IrSn_2_, IrCr, IrTi, and IrNi in 0.5 M H_2_SO_4_. **(C)** Geometric current density Tafel plot. **(D)** DLC-normalized specific activity. Reprinted (adapted) with permission from Strickler et al. (2019). Copyright 2019 American Chemical Society.

Molecular transition metal-based catalysts are of particular interest for water oxidation. Brudvig and co-workers specifically focused on Mn, Ru, Ir, Fe, and Co (Blakemore et al., 2015).Ru, Ir, Pd, and Pt also show good catalytic activity toward OER. Jaramilo and co-workers have also revolutionized the use of Ru- and Ir-based electrocatalysts for efficient oxygen evolution reactions (Seitz et al., 2016; Strickler et al., 2019; Hubert et al., 2020); (Figure 6). To decrease the usage of precious metals, it is very important to rationally design specific topological and electronic structures of catalysts to achieve satisfactory activity and stability(Xie et al., 2019). Noble metals and their alloys, oxides, and composites are among the most-studied catalysts for water oxidation for their excellent performance for OER. Experimental evidence shows that Ir and Ru are more active towards OER than Pt or Pd (Ru > Ir > Pd > Pt)(Jiao et al., 2015). Ir and Ru are recognized as the state-of-the-art water oxidation electrocatalysts with relatively low overpotential and Tafel slope as well as superior stability (Danilovic et al., 2014), though Ru has comparative less stability. The 3D superstructure with Ir was recently developed containing ultrathin nanosheets, and the excellent catalytic activity of the electrode was seen in both acidic and basic media, even better than Ir NPs (Pi et al., 2016). Considering the oxides, iridium oxide (IrO_2_) and ruthenium oxide (RuO_2_) are the most promising for OER (Ledendecker et al., 2019), though RuO_2_ NPs have slightly higher intrinsic and mass OER activities than IrO_2_. Recently, the activity trends of noble metal-based materials have been identified: Ru > Ir ≈ RuO_2_ > IrO_2_, while the dissolution trend is as follows: Ru >> Ir > RuO_2_ >> IrO_2_. Thus, oxides have more potential applications with better stability and good activity here as metal dissolution is very much higher in both the acidic and basic medium. In spite of having substantial applications as efficient OER electrocatalysts, these materials suffer from high cost, unsatisfactory long-term stabilities, and limited compositions or morphologies that further calls for through investigation in designing robust noble metal-based materials for OER electrocatalysis (Shi et al., 2019).

### Earth-Abundant Transition Metals

**FIGURE 7:**
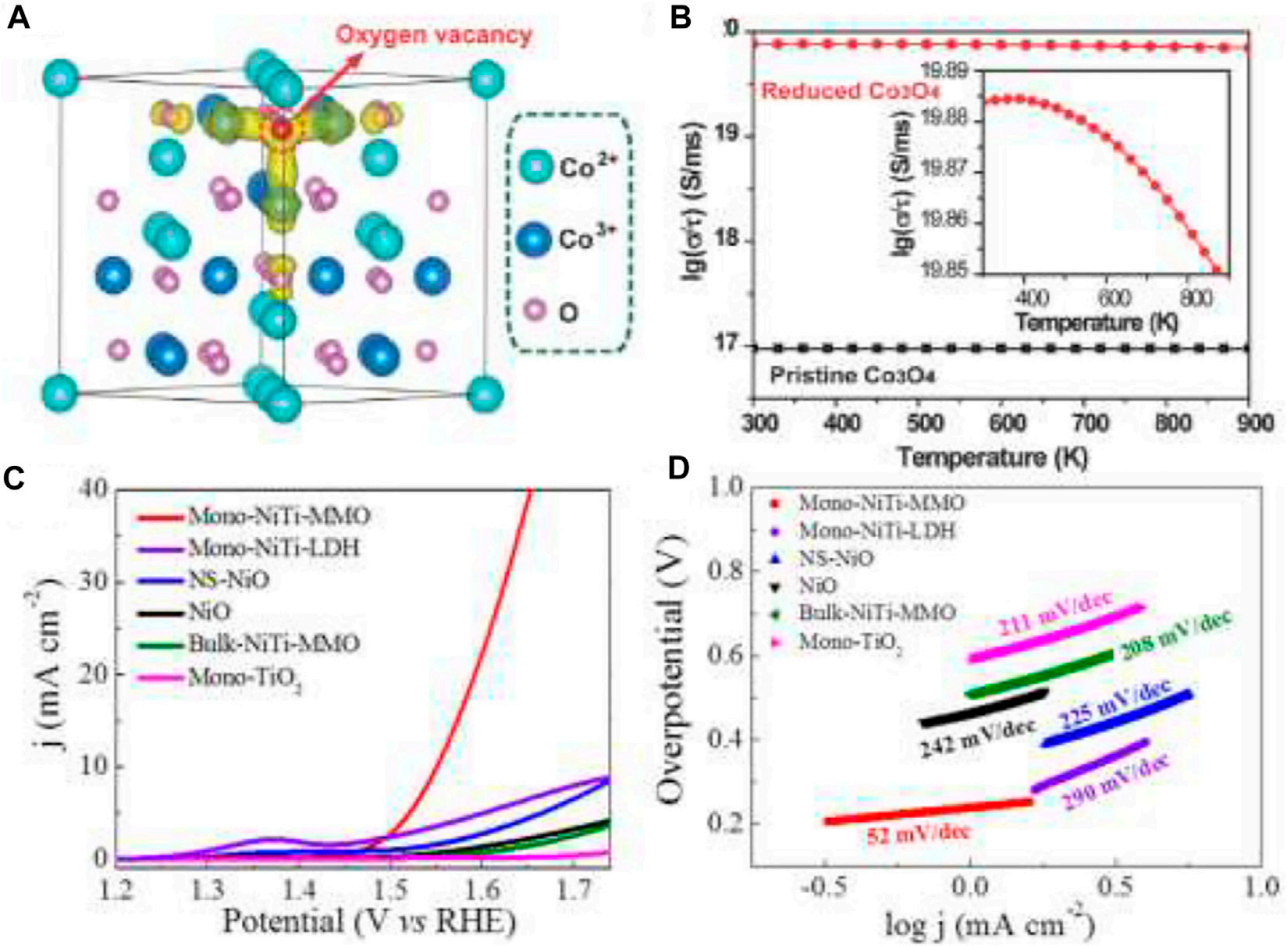
**(A)** Partial charge density of the reduced Co_3_O_4_. The states of Vo^2+^ are displayed in yellow. The iso-surface levels are 0.037 e Å^−3^. **(B)** Calculated conductivity for the reduced Co_3_O_4_ and the pristine Co_3_O_4_. Inset: An expansion of the plot of reduced Co_3_O_4_ (Wang et al., 2014) OER performance; **(C)** linear sweep voltammetry (LSV) curves; **(D)** corresponding Tafel plots (Zhao et al., 2016).

Earth-abundant transition metal oxides have low costs, plentiful sources, and good corrosion resistance, and that is why their application has increased so much in the field of electrocatalysts (OER/HER/ORR). Their multivalent oxidation states (M^+2/+3/+4^) make them excellent candidates for OER by providing the active sites for reaction (Risch et al., 2015). The OER activities of these materials are highly dependent on composition, oxidation state, morphology of the surface oxygen binding energy, the 3d electron number of transition metal ions, and the enthalpy for the lower- to higher-oxide transition (Yang et al., 2014). The intrinsic activity of individual active sites is dictated by their electronic structure. Cationic (e.g., Ni, Fe, Co, Sn) and anionic (e.g., O, S, OH) regulation is revealed to be a promising method for transition metal compounds to alter the electronic structure and generate high activity (Tang et al., 2018). During OER, phase conversions of Co-based materials, such as oxides to hydroxides or oxohydroxides, play a major role besides the multiple valence states. Wang et al. specified that the activity of Co_3_O_4_ largely depends on the morphology, surface area, and oxidation state (Wang et al., 2014). Mesoporous Co_3_O_4_ nanowires have a high surface area leading to more active sites, and the oxygen vacancies in it enhance the electrical conductivity due to the formation of new states through the delocalization of the Co–O bonds (Figures 7A,B).

Similar to cobalt oxide, great efforts have been made to improve the activity of NiO by tuning the particle size, surface area, and surface micro-structure (Han et al., 2016a) (Figures 7C,D). Ni^3+^ is very active as the higher oxidation state for OER. Ultra-small crystalline NiO NPs are fabricated using solvothermal reaction to form Ni^3+^ on the surface due to the small particle size of NiO. Therefore, the reactivity normally increases with the surface energy in the order {101} > {113} > {100} for NiO facets (Zhao et al., 2016). The oxides of manganese are other candidates to display excellent water oxidation activity. The catalytic activity again is dependable on the crystallographic structure, morphologies, pore structure, and chemical compositions. The good OER activity of manganese oxides in alkaline solution (Gorlin and Jaramillo, 2010) is mainly attributed to the nanostructured nature expediting the occurrence of suitable Mn_x_O_y_ active sites at the related potentials. The order of activity of different crystal structures of manganese oxides is α -MnO_2_ > amorphous MnO_2_ > β -MnO_2_ > α -MnO_2_ (Meng et al., 2014). Mixed metal oxides, like NiCo_2_O_4_, are very efficient for electrocatalysts for high electrical conductivity and excellent electrochemical activity. Hollow, porous, and complex 3D structures (NiCo_2_O_4_) also have a very positive effect on the water oxidation activity (Han et al., 2016b) due to the penetration of the electrolyte and sufficient contact area between the electrolyte and reactants. Mixed Ni–Fe electrocatalysts are also widely studied for electrocatalytic activity in alkaline solution (Görlin et al., 2016). LiCoO_2_ has also attracted considerable attention in OER for its stability and good activity, cubic NPs of which exhibit great water oxidation ability in neutral and basic electrolytes in all means (Chen et al., 2016).

### Hydroxides

Hydroxides generally have cationic brucite-like layers separated by charge balancing interlayer anions, and this open structure helps in fast diffusion with a rapid proton-coupled electron structure. Thus, the easy availability of catalytic active sites gives rise to a good electrocatalytic activity. The layered structure also allows the intercalation of water molecules and anions, providing better bulk redox activity. The typical hydroxide of water oxidation is Ni(OH)_2_{ α *and* β }. For better stability and activity in alkaline solution, α -Ni(OH)_2_ is more suitable for this purpose than β -Ni(OH)_2_. Subbaraman et al. have found the following trend of 3d metal hydroxides with defined morphologies and stoichiometric ratio: Mn < Fe < Co < Ni (Subbaraman et al., 2012).

### Perovskites

Perovskites, having the general formula ABO_x_ with alkaline earth or rare earth metals (A) and transition metals (B) in their composition, are very important for OER due to flexibility in their composition and structure (Zhu et al., 2016). CoSn(OH)_6_ perovskite nanocubes can be electrochemically carved in such a way to make it extremely active for OER (Song et al., 2016).Crystal vacancies play a significant role in the construction of active catalysts. The activity of transition metals shows a volcano-shape necessity on the tendency of 3d electron and e_g_ symmetry in the oxide of the transition metal. The highest OER activity is expected at e_g_ occupancy very close to unity with high covalency of metal-oxygen bonds. Fabricated Ba_0.5_Sr_0.5_Co_0.8_Fe_0.2_ *O*_3−_ (BSCF) obtained higher OER activity than IrO_2_ in an alkaline medium (Suntivich et al., 2011).

### Metal Phosphides/Phosphates

Transition metal (Ni, Co, Mn) phosphates are more stable in oxidative environments than the oxides mainly because of their flexible structural natures as well as different orientations of the accessible phosphate groups in their crystals (Zhang et al., 2016). The main participant in this category is Co-based phosphates/phosphides (CoP, Li_2_CoP_2_O_7_, Na_2_CoP_2_O_7_, NaCoPO_4_ and LiCoPO_4_). Serving as a working electrode, well-defined mesoporous CoP nanorod arrays on Ni foam display good OER activity (Zhu et al., 2015). The electrode is found to have excellent electric interconnection along with better mass transportation. Cations and anions significantly optimize the electrocatalytic activity by enlarging the active sites in the electrode and have important roles in overall water splitting (Duan et al., 2016). Thin films work more efficiently than pasting catalyst materials into electrodes or fabricating directly into some support such as Ni foam or carbon cloth because the deactivation by mechanical shredding or poisoning from the supporting materials can be prevented in this way. For example, Yang et al. made a diverse-phased porous Co phosphide/phosphate thin film that functionalizes directly as a working electrode. Similar to CoP, Mn and Ni based phosphides (NiP, Ni_2_P, MnP, CoMnP, etc.) are also studied for their water oxidation activities (Yang et al., 2015). Carbon-coated porous NiP (Yu et al., 2016) exhibits much better stability and excellent activity by improving the conductivity as well as charge transfer ability than MnP. In addition, MnP is not stable under oxidizing conditions due to the oxophilic nature of Mn. Recently, their performance are enhanced to much extent through the modulation of porosity, morphology, and combining with different materials (Stern et al., 2015). Furthermore, electrochemical studies show that oxygen evolution from Co-Pi prepared by electrodepositions from Co^2+^ solutions in phosphate electrolytes involves a turnover-limiting chemical step that is preceded by a one-electron, one-proton PCET equilibrium step (Surendranath et al., 2010).

### Carbonaceous Materials

Carbon-based materials, such as carbon nanotubes (CNTs), nanocarbons, graphenes, and mesoporous carbons, have been found very much active for electrocatalytic oxygen evolution reactions. They have a large specific surface area and excellent electrical conductivity along with good stability, making them perfect candidates for OER. Their performance can be further enhanced by shaping the geometrical and electrical structure of the electrode and doping materials such as N, S, P, B, O, etc., in the electrode material. Carbon atoms, bonding to electronegative oxygen or nitrogen, are positively charged. These positively charged carbon atoms adsorb *OH* ^−^ through charge transfer and accelerate the recombination of intermediates such as *O*_2_^−^ and *O*_2_^2−^ to form oxygen gas and thus decrease the activation energy for the process. Basically, the rational control of the electronic structure, strong chemical synergetic effect, and carbon defects prompted by the external carbon atoms enhance the activity (Yu X. et al., 2016). In nitrogen-doped carbon nanomaterials, Zhao et al. reported that the active sites for OER activities were pyridine and quaternary N (Zhao et al., 2013). Co-doping in carbon materials is another significant method, and Chen et al. reported the synthesis of N, O co-doped graphene carbon nanotube (NG-CNT) hydrogen film, which shows high OER activity overtaking IrO_2_ (Chen et al., 2014) with robust stability in alkaline and acidic solutions. Yu et al. conducted the experiment with N, S co-doped graphite foam (NSGF), which is directly used as an electrode without any polymeric binder. It showed fast kinetics and lower overpotential than N-doped graphite foam in alkaline solutions (Yu X. et al., 2016). Ma et al. designed the fabrication of g-C_3_N_4_ nanosheets–carbon nanotubes to have a strong composite for water oxidation possessing high nitrogen content, easily tailorable stricture, and low cost (Ma et al., 2014a).

### Hybrid Nanostructure

**FIGURE 8:**
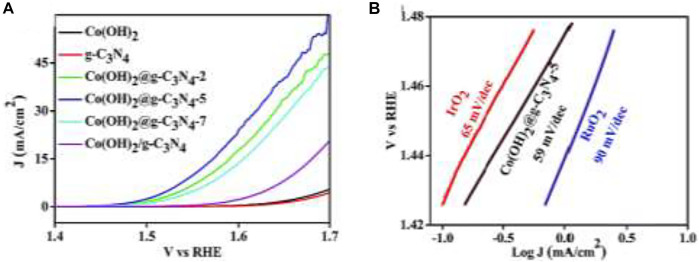
**(A)** LSV curves of g-C_3_N_4_, Co(OH)_2_/g-C_3_N_4_, Co(OH)_2_, and Co(OH)_2_@ g-C_3_N_4_ NWs and **(B)** Tafel slope of Co(OH)_2_@g-C_3_N_4-5_, RuO_2_, IrO_2_, and Pt/C (Tahir et al., 2016).

The hybrid materials used for water oxidation include carbon material-based, Graphene-based, g-C_3_N_4_-based, and many others for their high carrier mobility and long-term durability (Zhu et al., 2017). The interface should be strong between the catalyst materials and working electrode for high performance. Co_3_O_4_ nanosheets are deposited on carbon paper (Co_3_O_4_-CP), which shows high catalytic activity towards water oxidation in 0.5 M H_2_SO_4_ with an excellent overpotential of 370 mV and amazing stability. For increasing the interface-contact strength, Co_3_O_4_-carbon porous nanowire arrays are prepared through carbonization on the Cu foil (Ma et al., 2014b). Carbon-hybrid materials can also be made by layer double hydroxide, such as the fabrication of nanoplates of crystalline ultrathin Ni–Fe layered double hydroxide in multi-walled carbon nanotubes (Gorlin and Jaramillo, 2010). Nitrogen-doped hybrids based on phthalocyanines and porphyrins are also fabricated to boost the OER (Masa et al., 2014). High conductivity, large surface area, and atomic thinness make 2D carbon material graphene a fit substance to make composites with other transition metal oxides/hydroxides/sulfides for water oxidation OER. It can minimize the agglomeration of other nanoparticles to enhance the stability of the electrode. One such example of its use is crumpled graphene-cobalt oxide nanocrystal hybrids, which can fully utilize the catalytic surface area through the minimization of agglomeration of graphene sheets (Mao et al., 2014). Because of high nitrogen content and amazing stability together with graphene alike structure made g-C_3_N_4_ popular for hybrid OER electrocatalysts. Hybrid cobalt–hydroxide nanowires coated with graphitic carbon nitride nanosheets (Tahir et al., 2016) demonstrate a low overpotential of 320 mV at 10 mA *cm* ^−2^ for water oxidation (Figure 8), showing better capabilities than Co(OH)_2_, IrO_2_, RuO_2_, and g-C_3_N_4_. Core-branch hydroxysulfides like Co_2_NiS_2.4_(OH)_1.2_ also exhibit superior OER performance, with a remarkably low overpotential (279 mV required for 10.0 mA cm^−2^), a low Tafel slope (52 mV dec^−1^), and a favorable long-term stability(Wang et al., 2019). Spatially confined hybridization of nanometer-sized NiFe hydroxides into nitrogen-doped graphene frameworks is also found to exhibit superior OER activity (Tang et al., 2015).

### Photochemical Water Oxidation

**FIGURE 9:**
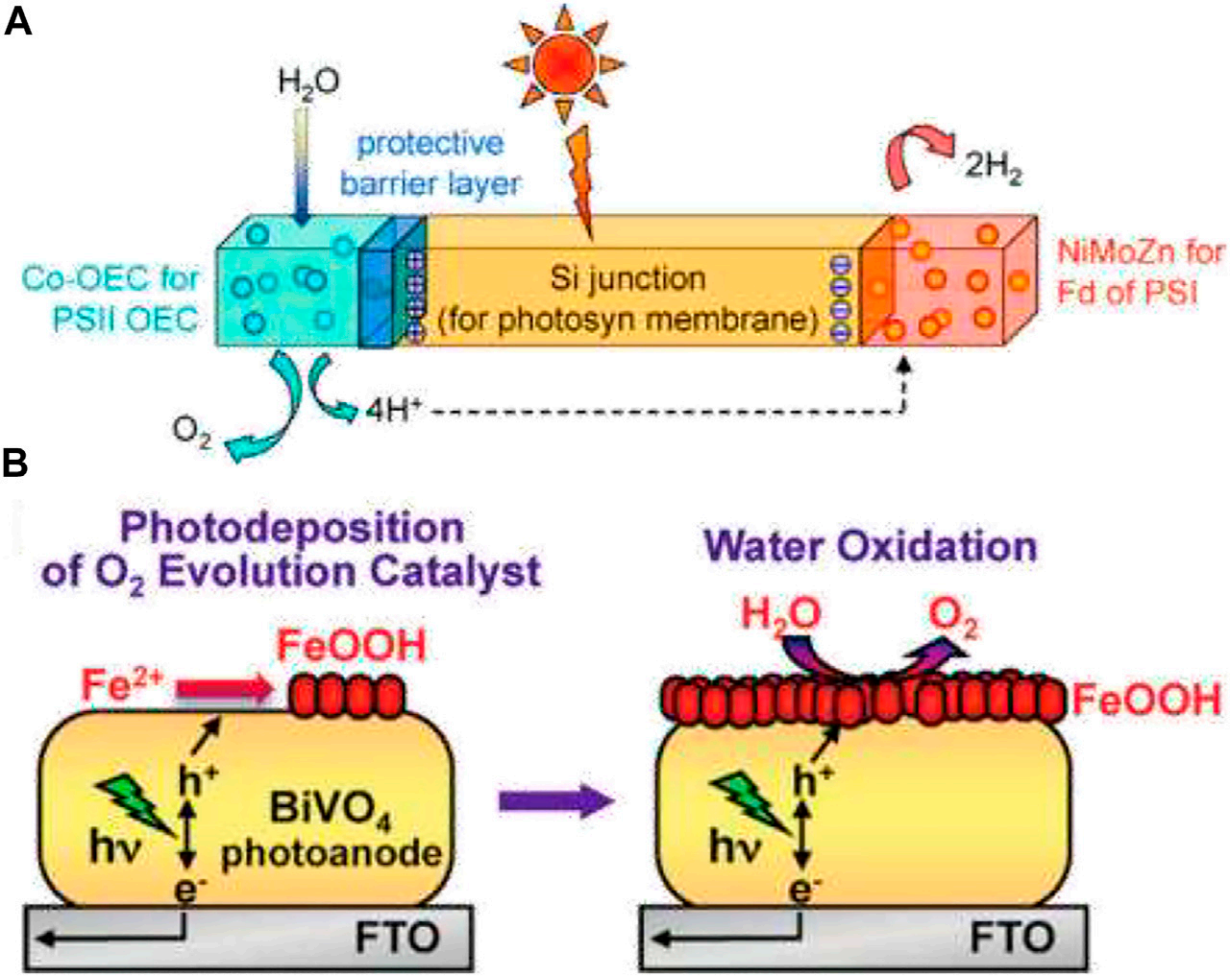
**(A)** Construction of an artificial leaf. The photosynthetic membrane is replaced by a Si junction, which performs the light capture and conversion to a wireless current. The oxygen-evolving complex and ferodoxin reductase of the photosynthetic membrane are replaced by Co-OEC and NiMoZn OER and HER catalysts, respectively, to perform water splitting. Reprinted (adapted) with permission from (Nocera, 2012). Copyright (2012) American Chemical Society. **(B)** Photo-oxidation of water by a bismuth vanadate photoanode coupled with an iron oxyhydroxide oxygen evolution catalyst. Reprinted (adapted) with permission from Seabold and Choi (2012). Copyright (2012) American Chemical Society.

The earliest reported semiconducting material for this purpose is TiO_2_ for PEC water oxidation, which can stably oxidize water without any surface modification (Wang et al., 2013b), but it generally absorbs in the UV region, thus making its solar conversion efficiency insufficient for practical use. For water oxidation, most of the active electrocatalysts need an overpotential of around 0.2 V (McCrory et al., 2013). Surface-active sites for hematite electrodes to produce O_2_ or H_2_O_2_ depend on the surface hole density. Amorphous and disordered catalysts, formed by electrodeposition or some low-temperature method, are more efficient for photocatalytic water oxidation due to their higher permissibility for the electrolyte. Dense catalysts form buried junctions, and the performance of the modified electrodes is then limited by Fermi-level pinning. Norcera et al. developed an electrodeposited “CoPi” catalyst for using it in various photoanodes due to the ease of deposition and high activity (Nocera, 2012); (Figure 9A). Fe doping in Co- and Ni-based OER electrocatalysts has emerged as the improved version for the problem of the low-bias performance of BiVO_4_ electrodes (Morales-Guio et al., 2016). The PEC performance of photoanodes is sensitive to the thickness of the catalyst on the electrode surface if the catalyst is charged during decomposition for nanostructured electrodes with large surface areas. FeOOH provides an example to show much better stability for both planar and nanoporous BiVO_4_ electrodes than Co-based catalysts (Seabold and Choi, 2012); (Figure 9B) as a thick layer of catalyst can increase significantly the chance for the forward electron transfer of electrons, resulting in a severe surface charge recombination. The recently developed champion for electrochemical water oxidation is ternary FeCoW oxyhydroxide, and its high catalytic activity is ascribed to a synergistic interplay between tungsten, iron, and cobalt (Zhang et al., 2016). Some other promising materials for photoelectrochemical water splitting include SnNb_2_O_6_, Ta_3_N_5_, BaTaO_2_N, Si, GaAs, CdTe, etc.

## Translating Academia to Industry: OM-Redox, an Oxygen Generating Device by Solaire Initiative Private Ltd

In recent years, water oxidation reaction and, more specifically, oxygen evolution reaction have become a hot avenue for translating knowledge from academia to industry because of its widespread industrial applications in water splitting, rechargeable metal–air batteries, and fuel cells. Research is being performed to develop efficient electrocatalytic systems for these reactions, which can meet the industrial requirements of current densities of ≥500 mA cm^−2^ at overpotentials of ≤300 mV with long-term stability (Yu et al., 2021). Of late, water electrolysis has emerged as a reliable and eco-friendly way to produce high purity H_2,_ which is an environmentally benign energy carrier and feedstock for diverse applications, ranging from chemical industry to transportation, and power sectors. Over the past few decades, Ir- and Ru-based oxide electrocatalysts have been widely used in industrial proton-exchange membrane water electrolyzers (Li et al., 2020). However, their high cost, as well as limited supply, restricts their applications, which makes non-precious-metal-based catalysts [e.g., oxides (Song et al., 2018) and (oxy)hydroxides (Yu et al., 2021) of first-row transition metals] great alternatives for the job. Among various electrochemical energy storage systems, metal–air battery presents very high theoretical energy density (e.g., the specific energy density of lithium–air battery is 40.1 MJ/kg, almost approaching to 40.6 MJ/kg of gasoline) (Gao et al., 2016). Li–air batteries have emerged as a potential replacement for Li-ion batteries in automotive applications. Research has been going on to develop novel material for core elements (porous carbon for air cathodes, catalysts, electrolytes, oxygen selective membranes, protected Li-metal) to make air-stable Li–air cells (Park et al., 2012). Efforts are also being made to develop bifunctional electrocatalysts to lower the overpotential of ORR and OER during the discharging and charging processes, which will help us to understand the full potential of this technology which is still arguably in its infancy (Vazhayil et al., 2021). A fuel cell is an electrochemical device that converts chemical energy to electrical energy. Among various available types of fuel cells, solid oxide and reversible fuel cells are more popular because of their ability of energy storage and fuel resurge, respectively. OER, being a part of the water electrolysis process, becomes important for developing these platforms. On the other hand, electrochemical water oxidation reaction also presents a feasible option to produce highly pure oxygens for medical and industrial purposes. According to WHO, during the ongoing COVID-19 pandemic, more than half a million COVID-19 patients in low and low middle-income countries estimated to need oxygen treatment every day (WHO, 2021). There are several methods commonly employed in medical oxygen productions (e.g., cryogenic distillation method, vacuum pressure swing adsorption, ceramic air separation technology, etc.), but all of them suffer from disadvantages such as its high cost, high energy consumption, contamination, or low production of pure oxygen. This makes electrochemical methods producing oxygen attractive because of their economical approach and eco-friendly nature. Pushkarev et al. reported an electrochemical oxygen pump (concentrator) with a solid polymer electrolyte which provides efficient *in situ* production of highly pure oxygen at a twice lower energy consumption as compared to the water electrolyzer with a solid polymer electrolyte (Pushkarev et al., 2020).

**FIGURE 10:**
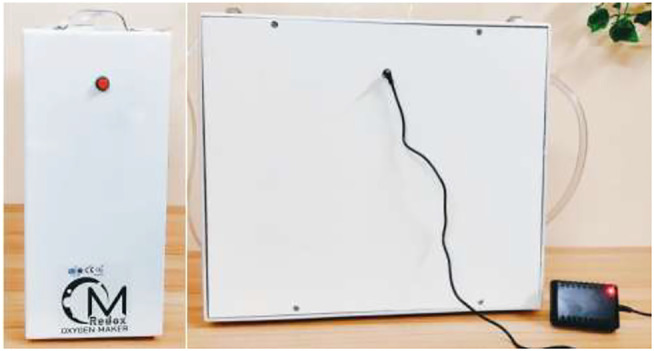
Product oxygen maker, OM REDOX; the left and right sides present the front and side view of the prototype.

We at Solaire with our product OM-REDOX (Oxygen Maker REDOX) have addressed the issue of portable oxygen supply at a very low cost (Figure 10). We have designed a device, which can produce oxygen by using water. The oxygen production rate is in the range of 3–5 L per minute (Lpm) and can be enhanced to 20–30 Lpm. Our patented product design and catalyst design (Patent file number: 202131020605) have been developed that can carry out water splitting under specially designed and fine-tuned patented condition. The process of biological water splitting bears resemblance to water splitting in which a chemical potential difference is used to split water. We have made several experiments to confirm with which catalyst the production of oxygen is the highest, and we have rigorously tested the quality of the catalyst and even ratified it with a hypoxia curing mouse model as well. The product uses electricity, which activates the catalyst in a sophisticated circuitry developed in-house by us. As we switch on the electricity oxygen is produced. The key process in our product lies in integration of mechanical, electrical, and chemical engineering with catalyst design science using easily sourced components and starting materials, rendering the product scalable and easy to manufacture in large scale. The simplest combination of circuitry and mechanical and chemical engineering principles used in this product reduces the production time and makes it easy to build and deliver to millions in need. The product OM REDOX is a direct demonstration of the translation of an academic result to a product in the market.

## Conclusion

The kinetics behind OER is quite sluggish, which makes water oxidation a difficult reaction to proceed. This necessitates the need for a catalyst and the optimal design of the electrode that can run the reaction at lower potential by overcoming the high activation energy barrier. In recent times, the electrocatalytic OER activity has been slightly improved due to the understanding of the scaling relation between HO* and HOO*. However, the targeted catalyst design should incorporate features of breaking the HOO* and HO* scaling and independently optimizing the adsorption energies of various intermediates (Kulkarni et al., 2018). Understanding the catalytic cycle with minute mechanistic details is very important to design efficient catalysts. It is very difficult to understand and control various competing redox mechanisms in OER. Hence, using various *in-situ* characterization techniques (e.g., femtosecond spectra, near-atmospheric X-ray photoelectron spectra (NAP-XPS), near-atmospheric scanning tunneling microscopy (NAP-STM), *in situ* X-ray absorption spectroscopy (XAS), and *in situ* diffuse reflectance infrared Fourier transform (DRIFT) spectra, corroborated with DFT computations, etc.) to capture a complete overview of the phase transformations, valence state changes, morphological variations, etc., under OER conditions is necessary (Suen et al., 2017). Search for non-noble metal-based OER catalytic systems has also gained momentum because of high cost, unsatisfactory long-term stabilities, and limited compositions or morphologies of noble-metal-based systems. This has been discussed in detail in this review. Efforts to find efficient OER systems are not only focused on developing efficient catalytic materials, and it also finds its ways to efficient electrode and cell design strategies. Modifying the electrode surface by tuning the active catalytic sites, surface facets, interfaces has been in the forefront of electrode designs. However, uneven current and potential distributions have been the bottleneck problem to these approaches, which needs further attention. OER is used to make devices such as metal–air batteries and mainly production of hydrogen through water electrolysis. As for the process and application, sustainable energy utilization in OER is vital for its further applications, specifically using the cost-effective triboelectric nanogenerator. This advanced development can utilize sustainable mechanical energy such as wind, rain, etc., to obtain sustainable self-powered OER systems in the near future, and this self-powered approach is very perspective regarding the future goal of green energy generation. The water oxidation process takes place in nature on its own in plants photosystem II to provide protons and electrons for the photosynthesis process, and thus, oxygen gas is released in the atmosphere. Furthermore, water oxidation can be coupled with organic reactions, like CO_2_ reduction to formic acid and formaldehyde for synthesis purposes of important organic compounds. Therefore, water oxidation has immense importance as the green process for further futuristic aspects and improvement of the electrode design (geometry and electrocatalyst) will help to achieve more complex purposes in a sustainable way.
